# Use of a dual genetic system to decipher exocrine cell fate conversions in the adult pancreas

**DOI:** 10.1038/s41421-022-00485-0

**Published:** 2023-01-03

**Authors:** Huan Zhao, Xiuzhen Huang, Zixin Liu, Liang Lai, Ruilin Sun, Ruling Shen, Yan Li, Lingjuan He, Wenjuan Pu, Zan Lv, Yi Li, Ximeng Han, Xiuxiu Liu, Bin Zhou

**Affiliations:** 1 State Key Laboratory of Cell Biology, Shanghai Institute of Biochemistry and Cell Biology, Center for Excellence in Molecular Cell Science, Chinese Academy of Sciences, University of Chinese Academy of Sciences, Shanghai, China; 2 Shanghai Model Organisms Center, Inc., Shanghai, China; 3 Shanghai Laboratory Animal Research Center, Shanghai, China; 4 School of Life Sciences, Westlake University, Hangzhou, Zhejiang China; 5 School of Life Science and Technology, ShanghaiTech University, Shanghai, China; 6 Key Laboratory of Systems Health Science of Zhejiang Province, School of Life Science, Hangzhou Institute for Advanced Study, University of Chinese Academy of Sciences, Hangzhou, Zhejiang China

**Keywords:** Transdifferentiation, Regeneration

## Abstract

Unraveling cell fate plasticity during tissue homeostasis and repair can reveal actionable insights for stem cell biology and regenerative medicine. In the pancreas, it remains controversial whether lineage transdifferentiation among the exocrine cells occur under pathophysiological conditions. Here, to address this question, we used a dual recombinase-mediated genetic system that enables simultaneous tracing of pancreatic acinar and ductal cells using two distinct genetic reporters, avoiding the “ectopic” labeling by Cre-loxP recombination system. We found that acinar-to-ductal transdifferentiation occurs after pancreatic duct ligation or during caerulein-induced pancreatitis, but not during homeostasis or after partial pancreatectomy. On the other hand, pancreatic ductal cells contribute to new acinar cells after significant acinar cell loss. By genetic tracing of cell proliferation, we also quantify the cell proliferation dynamics and deduce the turnover rate of pancreatic exocrine lineages during homeostasis. Together, these results suggest that the lineage transdifferentiation happens between acinar cells and ductal cells in the pancreatic exocrine glands under specific conditions.

## Introduction

The exocrine pancreas makes up the vast majority of pancreatic mass, which is mainly composed of acinar cells and ductal cells^1,2^. The pancreatic acinar cells can produce and release large amounts of digestive enzymes, including amylases, lipases and proteinases, while the ductal cells can deliver these enzymes to the duodenum^2^. Previous work showed that adult pancreatic acinar cells might be an important source for beta cell neogenesis^3,4^, a result that received much enthusiasm and attention in the field. The centroacinar cells are defined as the specialized terminal end duct epithelial cells and have been proposed as progenitors for endocrine and exocrine pancreas regeneration^5–7^. All pancreatic epithelial lineages are presumed to originate from the common multipotent pancreatic progenitor cells during embryonic development^1,8–10^. However, whether exocrine lineage conversions exist under pathophysiological conditions in the adult mammalian pancreas remains controversial.

Multiple pioneering genetic studies based on the Cre-loxP recombination system showed divergent results regarding the contribution of ductal cells to postnatal acinar cell fate. One study using *CAII-CreER* suggested that adult carbonic anhydrase II (*CAII*)-positive pancreatic ductal cells act as progenitors that give rise to new acinar cells^11^. Another study found that *Sox9*-positive pancreatic ductal epithelia are progenitors for acinar cells under physiological condition^12^. However, several studies using lineage tracing strategy found that the exocrine acinar cells regenerated mainly by self-replication^13–17^. In addition, two ductal cell lineage tracing studies suggested no contribution of ductal cells to acinar cells in adults^18,19^.

Whether acinar cells can, in contrast, contribute to ductal cell fate also remains controversial. Transdifferentiation from acinar to ductal cells (acinar-to-ductal metaplasia) was observed in severe chronic pancreatitis (CP) lesions in adult mice in vivo and also in human pancreatic acinar cells cultured in vitro, where some acinar cells lost the abundant zymogen granules and transformed into duct-like cells^15,20,21^. Pan et al. found that a small number of *Ptf1a*-positive acinar cells could be reprogrammed to generate ductal cells after pancreatic ductal ligation (PDL) in adults^13^. However, Desai et al. did not observe any acinar-to-ductal transdifferentiation during homeostasis and several injuries using *Elastase-CreER* mice^14^. Sangiorgi et al. identified a subpopulation of pancreatic acinar cells that did not contribute to other lineages under normal condition and after injuries^16^. In addition to these lineage tracing controversies in the adult pancreas, the proliferation dynamics of the pancreatic exocrine cell lineages remains uncharacterized.

The conventional Cre-loxP genetic lineage tracing system has its limitations, which may lead to the discrepancies in multiple previous studies^22,23^. The reliability of this strategy is mainly based on the promoter specificity. The weak expression of the promoter gene may drive Cre expression beyond the threshold of Cre-loxP recombination but below the general detection level of immunostaining, thus potentially leading to a misinterpretation of cell fate plasticity^24^. Our lab developed a dual recombinase-mediated genetic system which incorporates the Dre-rox recombination system to trace cellular fate at a higher resolution and investigated several topics of recent debate^23–29^.

In this work, pancreatic acinar cells and ductal cells are simultaneously labeled by two distinct genetic markers that enable indelible tracing of their cell fates based on the dual recombinase-mediated genetic system. With this approach, we demonstrate that pancreatic acinar cells convert to duct-like cells, but not vice versa, during inflammation-related injuries but not during homeostasis or after partial pancreatectomy. Formation of new acinar cells from ductal cells is observed after Diphtheria toxin (DT)-mediated acinar cell loss. Furthermore, we also applied a cell proliferation tracing system^28^ to record the cumulative proliferation of pancreatic acinar cells and ductal cells during tissue homeostasis. Our work provides a detailed fate map of pancreatic exocrine cells during tissue homeostasis and after injuries, potentially resolving the long-held controversies over cell fate plasticity of these cells.

## Results

### Generation of a dual recombinase-mediated genetic system to simultaneously trace pancreatic acinar and ductal cells

To simultaneously trace pancreatic ductal cells and acinar cells in the same mouse, we planned to use dual recombinases Cre and Dre^24,25,29^ to map the fate of ductal cells and acinar cells, respectively. The exclusive labeling technique circumvents the “unwanted” cells labeled by Cre recombinase and enables the more precise tracing of cellular fate of two distinct cell populations^23^. If one recombination occurs, the other recombination could not occur, which results in a competitive effect for the two recombinases^23^.

To delineate the cellular fate map of pancreatic ductal cells, we used the *CK19-CreER*^30^ mouse line and crossed it with a responsive reporter line, *R26-tdT* (*Rosa26-loxp-Stop-loxp-tdTomato*). We treated 7–8-week-old *CK19-CreER;R26-tdT* mice with tamoxifen for three times in 5 days, and collected pancreatic tissues 2 days after the last tamoxifen injection for analysis (Supplementary Fig. S1a). Immunostaining for tdTomato (tdT), CK19 and E-cadherin (E-cad) on tissue sections revealed that the majority of pancreatic ductal cells were labeled by *CK19-CreER*, while a small subset of acinar cells was also tdT^+^ (Supplementary Fig. S1b, c). This “ectopic” labeling of pancreatic acinar cells by purported ductal-specific Cre driver may possibly lead to false-positive results and data misinterpretation. Therefore, the previous conclusions about the contribution of pancreatic ductal cells in acinar cell generation based on ductal-specific Cre needs a re-assessment to more closely scrutinize whether a minority of acinar cells were also pre-labeled. In our examination of a Dre driver, we found that a *Tnni3-Dre* knock-in line efficiently labels pancreatic acinar cells but not ductal cells in adult pancreas, rendering virtually all acinar cells tdT^+^ in *Tnni3-Dre;IR1*^25^ mice (Supplementary Fig. S2).

**Fig. 1:**
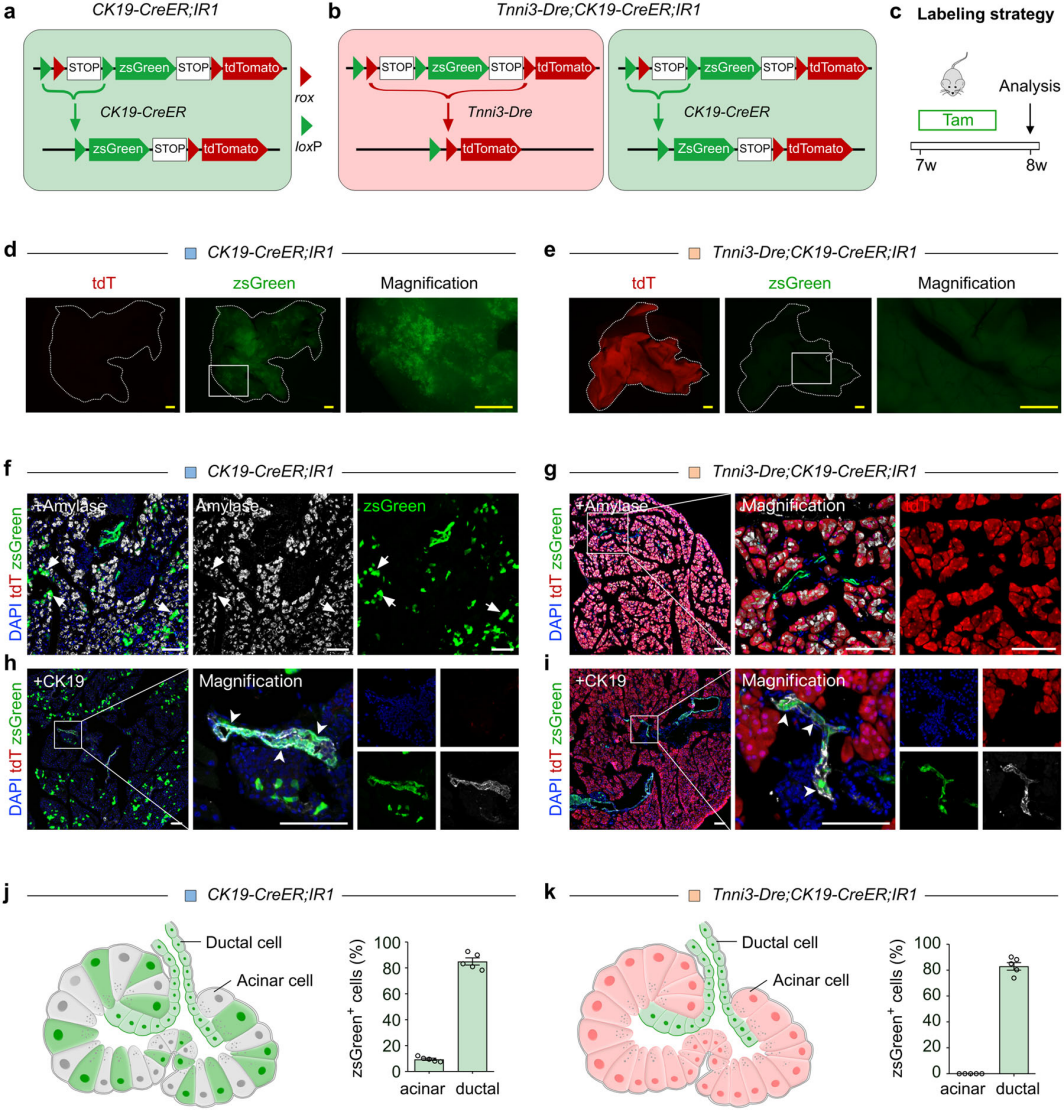
Genetic labeling of pancreatic exocrine ductal and acinar cells by a *Tnni3-Dre;CK19-CreER;IR1* strategy. **a** Schematic diagram illustrating the strategy for labeling pancreatic ductal cells by *CK19-CreER;IR1*. **b** Schematic diagram illustrating the strategy for labeling pancreatic ductal and acinar cells by *Tnni3-Dre;CK19-CreER;IR1* using a dual recombinase-mediated genetic approach. **c** Schematic diagram illustrating the experimental strategy for tamoxifen (Tam) induction and analysis. **d**, **e** Whole-mount fluorescent images of pancreas from *CK19-CreER;IR1* (**d**) or *Tnni3-Dre;CK19-CreER;IR1* (**e**) mice. **f**–**i** Immunostaining for tdT, zsGreen and Amylase (**f**, **g**) or CK19 (**h**, **i**) on pancreatic sections collected from indicated mice. Arrowheads, zsGreen^+^ ductal cells. Arrows, zsGreen^+^ acinar cells. **j**, **k** Schematic diagram illustrating the labeling strategy (left) and quantification of the percentage of ductal and acinar cells expressing zsGreen (right) by either *CK19-CreER;IR1* (**j**) or *Tnni3-Dre;CK19-CreER;IR1* (**k**) strategy. Data are means ± SEM; *n* = 5. Scale bars, yellow, 1 mm; white, 100 μm. Each image is representative of 5 individual samples.

Having validated *CK19-CreER* and *Tnni3-Dre* drivers, we then developed a genetic strategy (namely, *Tnni3-Dre;CK19-CreER;IR1*) to label pancreatic acinar cells and ductal cells simultaneously in the same mouse (Fig. 1a, b). Because of the interleaved recombinase-recognition sites in *IR1*, one recombination (for example, Dre-rox) could inherently remove one recombination site of the other system (loxP) to prevent subsequent recombination of another system (Cre-loxP).

Compared with the conventional tracing strategy involving *CK19-CreER;IR1* mice, constitutive Dre recombination of *IR1* blocks further inducible Cre-loxP-mediated recombination in acinar cells, thus excluding potential “ectopic” labeling of acinar cells by *CK19-CreER*. We treated 7-week-old *Tnni3-Dre;CK19-CreER;IR1* mice with tamoxifen for three times in 5 days, and collected pancreatic tissues for analysis 2 days after the last tamoxifen injection (Fig. 1c).

Whole-mount fluorescent imaging of the pancreas from *CK19-CreER;IR1* mice showed a readily detectable zsGreen signal, while pancreas from *Tnni3-Dre;CK19-CreER;IR1* mice exhibited mainly a tdT-positive signal with weak zsGreen activity (Fig. 1d, e). Immunostaining for tdT, zsGreen and the pancreatic ductal cell marker CK19 or the pancreatic acinar cell marker Amylase on tissue sections derived from the tamoxifen-treated *Tnni3-Dre;CK19-CreER;IR1* mice showed that virtually all acinar cells were tdT^+^ and the majority of pancreatic ductal cells were zsGreen^+^ (Fig. 1f–i; Supplementary Fig. S3). Broad zsGreen expression in ductal cells (85.06% ± 2.75%) and partly in acinar cells (9.53% ± 0.81%) was detected in tamoxifen-treated *CK19-CreER;IR1* mice (Fig. 1j). In contrast, no zsGreen^+^ acinar cells (0.00% ± 0.00% in acinar cells) were detected in *Tnni3-Dre;CK19-CreER;IR1* mice, while 83.51% ± 2.92% of ductal cells were zsGreen^+^ (Fig. 1k).

We barely found tdT^+^zsGreen^+^ double positive cells in the pancreas of *Tnni3-Dre;CK19-CreER;IR1* mice (Supplementary Fig. S4). As the dual recombinase-recognition sites were interleaved in *IR1*, recombination induced by one system could remove the recognition site of the other recombinase system, thus tdT and zsGreen signals are designed to be expressed exclusively in cells in this system. To further validate the dual recombination system, we also crossed *R26-iCre* with *Tnni3-Dre;IR1* mice. We treated *Tnni3-Dre;R26-iCre;IR1* mice with Doxycycline to enable expression of zsGreen in other non-acinar cell lineages in pancreas. Immunostaining for tdT and zsGreen on pancreatic sections revealed that no cells co-expressed tdT and zsGreen simultaneously (Supplementary Fig. S5). In examination of pancreas from *Tnni3-Dre;CK19-CreER;IR1* mice and *Tnni3-Dre;R26-iCre;IR1* mice by immunostaining, we did not find tdT expression in ductal cells and other non-epithelial lineages, such as endothelial cells, smooth muscle cells, fibroblasts or lymphatic endothelial cells (Supplementary Figs. S6, S7). In summary, we developed a dual recombinase-mediated genetic system marked by tdT^+^ acinar cells and zsGreen^+^ ductal cells specifically and simultaneously in the mouse pancreas.

### The homeostatic pancreas does not exhibit lineage transdifferentiation between ductal and acinar cells

**Fig. 2:**
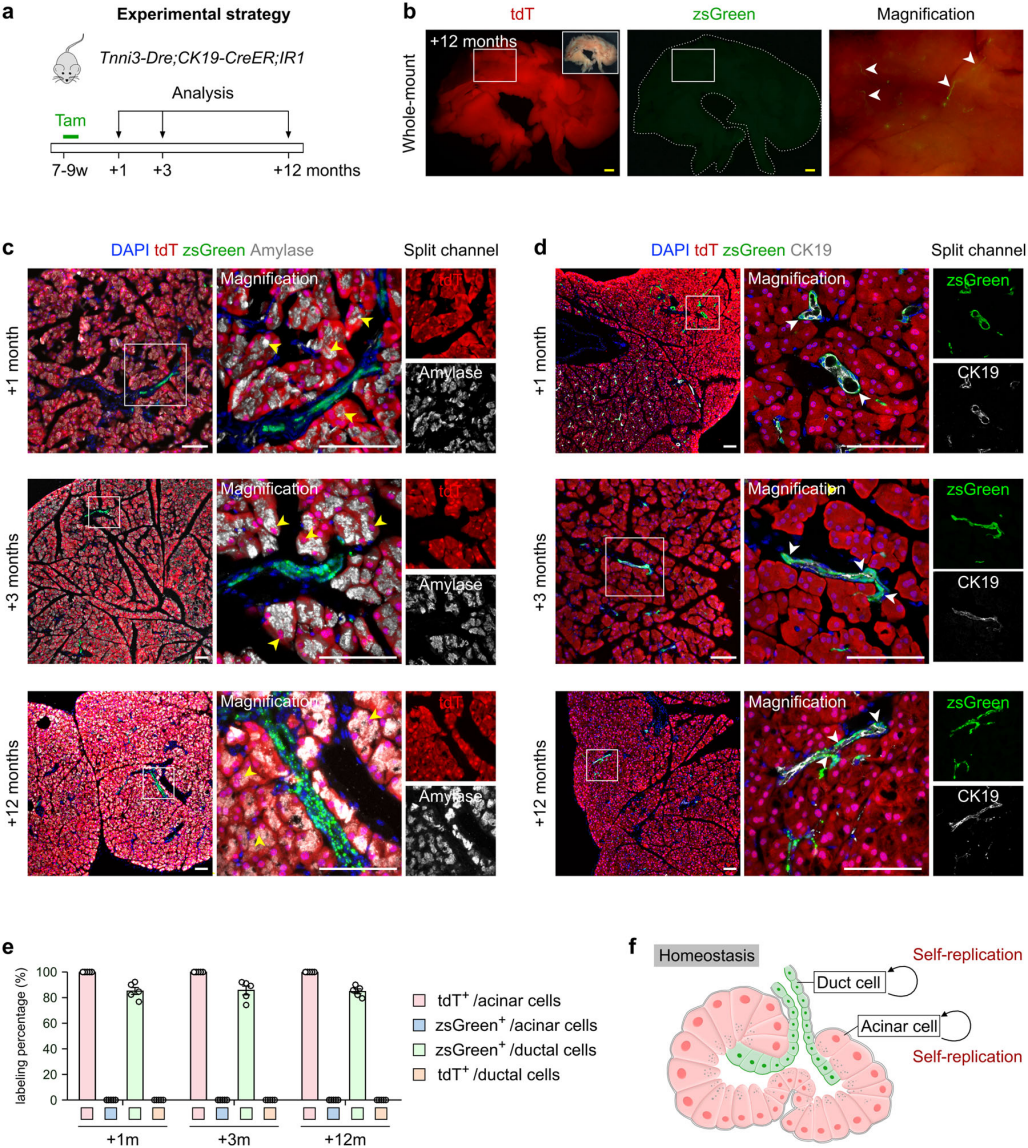
The pancreas does not exhibit lineage transdifferentiation between ductal and acinar cells during physiology. **a** Schematic diagram illustrating the experimental strategy for Tam-induced lineage tracing and analysis. **b** Whole-mount bright-field and fluorescent images for zsGreen and tdT expression in pancreas from *Tnni3-Dre;CK19-CreER;IR1* mice after 12 months of tracing. **c**, **d** Immunostaining for tdT, zsGreen and Amylase (**c**) or CK19 (**d**) on pancreatic sections collected from indicated time points. Yellow arrowheads, tdT^+^ acinar cells. White arrowheads, zsGreen^+^ ductal cells. **e** Quantification of the percentages of acinar cells expressing tdT or zsGreen and ductal cells expressing tdT or zsGreen from *Tnni3-Dre;CK19-CreER;IR1* mice at indicated time points. Data are means ± SEM; *n* = 5. **f** Schematic diagram illustrating that the pancreatic exocrine gland does not exhibit lineage transdifferentiation during homeostasis. Scale bars, yellow, 1 mm; white,100 μm. Each image is representative of 5 individual samples.

To test whether ductal cells contribute to acinar cells, or vice versa, under homeostatic conditions, we treated 7–9-week-old *Tnni3-Dre;CK19-CreER;IR1* mice with tamoxifen, followed by tissue analyses at 1, 3, or 12 months after the last injection (Fig. 2a). We preformed whole-mount fluorescent imaging of pancreas at the 12-month time point and found tdT^+^ signals throughout the entire pancreas coupled with sporadic zsGreen^+^ signals (Fig. 2b). Immunostaining for tdT, zsGreen, CK19 or Amylase on pancreatic tissue sections revealed that all acinar cells expressed tdT but not ZsGreen, while pancreatic ductal cells expressed ZsGreen but not tdT (Fig. 2c–e). These fate-mapping results suggest that during homeostasis in the adult mouse pancreas, new ductal cells and acinar cells mainly arise from self-duplication rather than lineage transdifferentiation (Fig. 2f), in line with previous results using other lineage tracing approaches^13–19^.

**Fig. 3:**
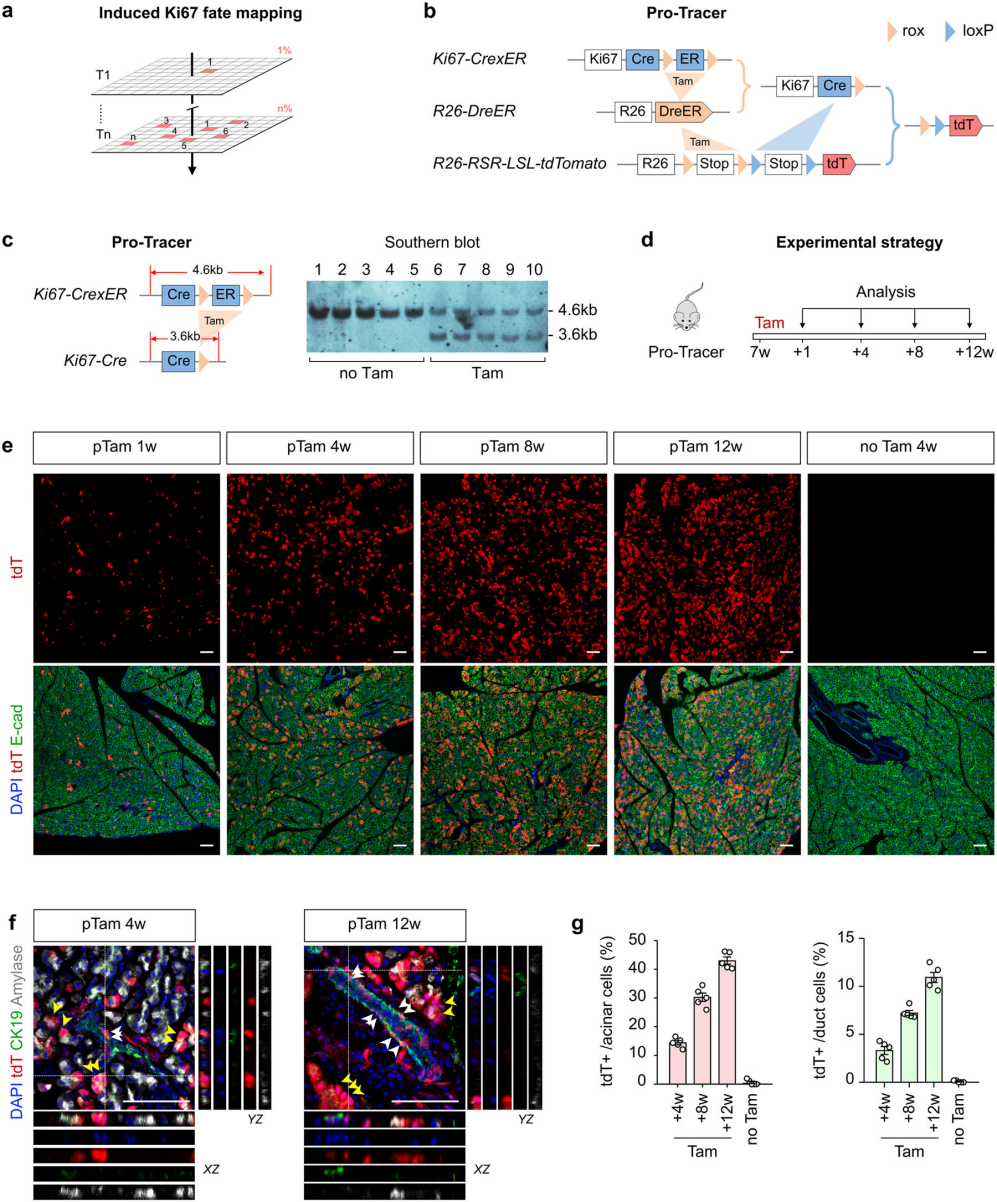
Genetic tracing of pancreatic acinar and duct cell proliferation during physiology using ProTracer system reveals cellular turnover dynamics. **a** Schematic diagram illustrating the recording of Ki67^+^ cells (tdT) from Time 1 (T1) to Time n (Tn) in pancreas. Each red square denotes a *Ki67*-expressing cell and progeny. **b** Schematic diagram illustrating the strategy for seamless recording of cell proliferation using ProTracer system. Tam-induced Dre/rox recombination switches *Ki67-CrexER* into *Ki67-Cre*, thus primes the mice to seamlessly record cell proliferation events. **c** Schematic diagram illustrating the strategy to detect floxed ER DNA excision in pancreas using Southern blotting of pancreatic DNA from Tam- or corn oil-treated (no Tam) ProTracer mice. **d** Schematic diagram illustrating the experimental strategy for Tam injection and post-treatment analysis. **e**, **f** Immunostaining for tdT and E-cad (**e**) or CK19 and Amylase (**f**) on pancreatic sections prepared from the indicated ProTracer mice. White arrowheads, tdT^+^ duct cells. Yellow arrowheads, tdT^+^ acinar cells. **g** Quantification of the percentage of duct and acinar cells expressing tdT by ProTracer mice from the indicated time points. Data are means ± SEM; *n* = 5. Scale bars, 100 μm. Each image is representative of 5 individual samples.

Based on our findings above, we then employed the genetic system for tracing cell proliferation, Protracer^28^ (*R26-DreER;Ki67-CrexER;R26-RSR-LSL-tdT*), in the current study to quantitatively measure the self-renewal of two exocrine lineages (Fig. 3a; Supplementary Fig. S8). In this design, one pulse of tamoxifen induces Dre/rox recombination and converts *Ki67-CrexER* into a *Ki67-Cre* genotype, thus permitting continuous recording of cell proliferation events in vivo (Fig. 3b). Southern blotting of pancreatic tissue genomic DNA showed that the switching rate of *Ki67-CrexER* to *Ki67-Cre* was ~50% (Fig. 3c). We then treated Protracer mice with tamoxifen and analyzed their pancreas at 4, 8 and 12 weeks after induction (Fig. 3d). Immunostaining for tdT, E-cad, or CK19 and Amylase on pancreatic sections showed that tdT signal gradually increased over time (Fig. 3e, f). Quantitatively, the percentage of Amylase^+^ acinar cells expressing tdT were 14.44% ± 0.75%, 30.28% ± 1.46%, 43.07% ± 1.27% at the 4-, 8-, and 12-week time points, respectively (Fig. 3g), suggesting a turnover rate of homeostatic acinar cells of ~7.18% per week (the switching rate is about 50%). The percentage of CK19^+^ pancreatic ductal cells expressing tdT were 3.32% ± 0.42%, 7.23% ± 0.26%, 10.92% ± 0.53% at the 4-, 8-, and 12-week time points, respectively (Fig. 3g), suggesting a turnover rate of homeostatic pancreatic ductal cells of ~1.82% per week (the switching rate is about 50%). Collectively, the above data obtained using the Protracer system provided proliferation dynamics of acinar and ductal cells in the pancreas during normal physiology.

### The pancreas after pancreatectomy does not display lineage transdifferentiation between exocrine lineages

**Fig. 4:**
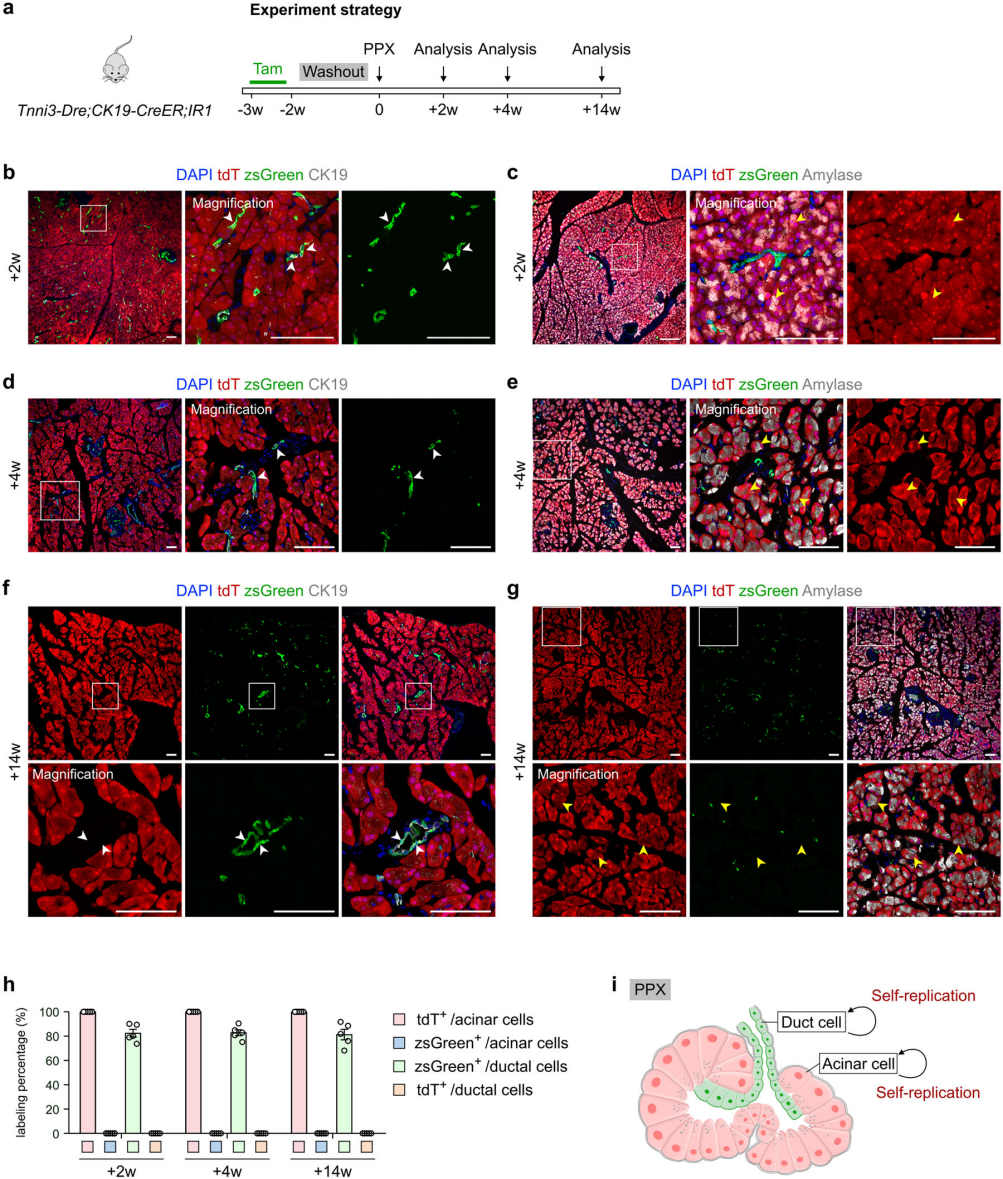
The pancreas does not exhibit lineage transdifferentiation between exocrine lineages after PPX. **a** Schematic diagram illustrating the experimental strategy for Tam induction, PPX injury and analysis. **b**–**g** Immunostaining for tdT, zsGreen and CK19 (**b**, **d**, **f**) or Amylase (**c**, **e**, **g**) on pancreatic sections collected from *Tnni3-Dre;CK19-CreER;IR1* mice at indicated time points. White arrowheads, zsGreen^+^ ductal cells. Yellow arrowheads, tdT^+^ acinar cells. **h** Quantification of the percentage of duct cells expressing zsGreen or tdT and acinar cells expressing tdT or zsGreen from *Tnni3-Dre;CK19-CreER;IR1* mice after PPX-induced injury. Data are means ± SEM; *n* = 5. **i** Diagram illustrating that pancreatic ductal and acinar cells do not exhibit lineage transdifferentiation after PPX. Scale bars, 100 μm. Each image is representative of 5 individual samples.

Previous studies reported that acinar cell formation and neogenesis were observed in the pancreas upon regeneration in models such as partial pancreatectomy (PPX)^11,31^. Next, we asked if lineage transdifferentiation occurs between pancreatic acinar cells and ductal cells under pathophysiological conditions known to stimulate exocrine cell proliferation. We treated *Tnni3-Dre;CK19-CreER;IR1* mice with tamoxifen. After 2 weeks of washout, we performed a 50% PPX to induce pancreatic tissue regeneration and collected pancreatic tissues at 2, 4, and 14 weeks after injury for analysis (Fig. 4a). Immunostaining for tdT, zsGreen, CK19 or Amylase on tissue sections revealed that all acinar cells were tdT^+^ZsGreen^–^ while the majority of ductal cells were ZsGreen^+^tdT^–^ (Fig. 4b–h). These data suggest no detectable lineage conversion between pancreatic acinar and ductal cells after PPX (Fig. 4i).

### Acinar-to-ductal transdifferentiation occurs after PDL and caerulein-induced pancreatitis

**Fig. 5:**
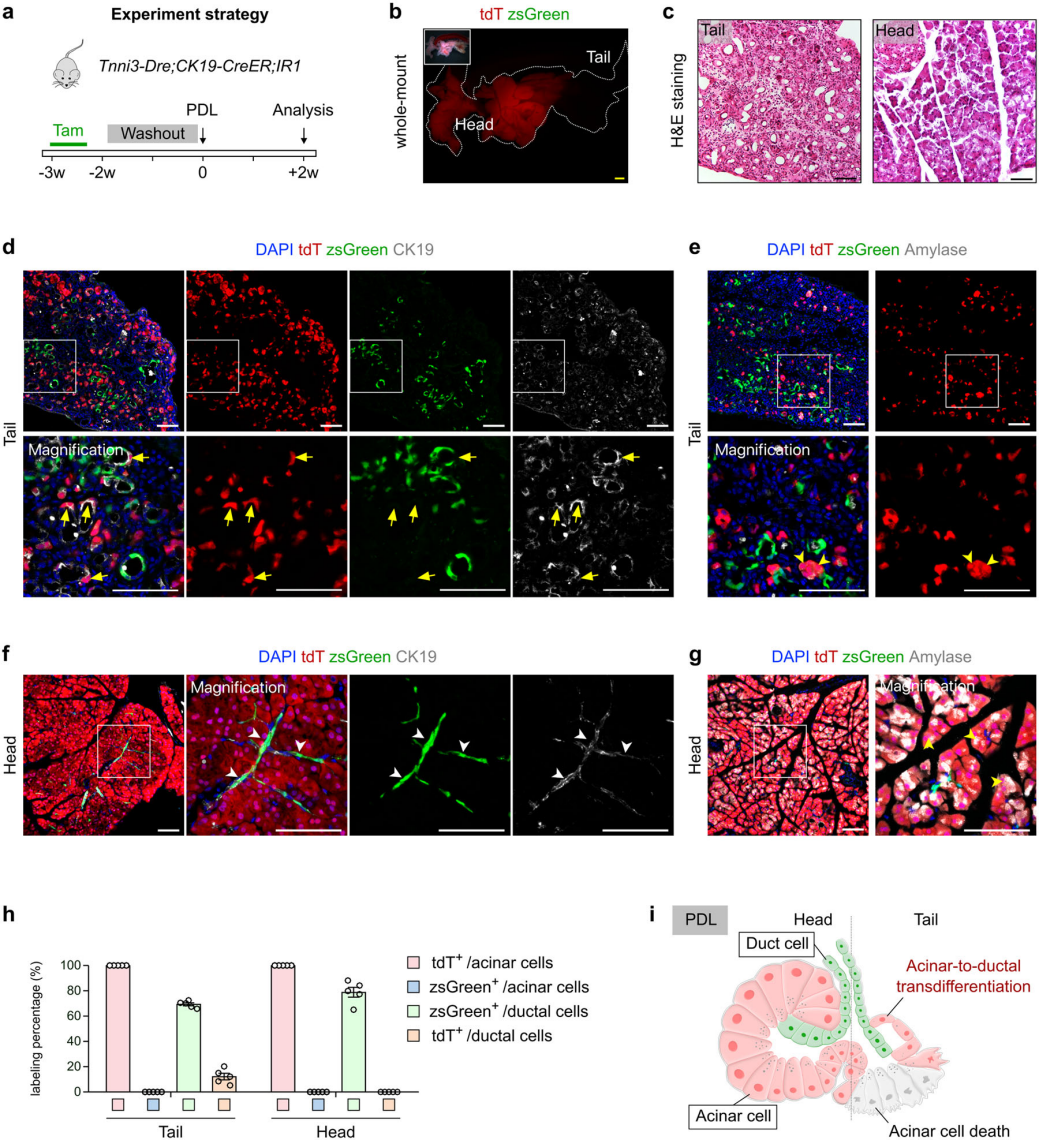
Acinar-to-ductal transdifferentiation occurs after pancreatic duct ligation-induced injury. **a** Schematic diagram illustrating the experimental strategy for Tam treatment, PDL-induced injury and analysis. **b** Whole-mount bright-field and fluorescent images showing zsGreen and tdT expression in the pancreas from *Tnni3-Dre;CK19-CreER;IR1* mice after PDL-induced injury. **c** H&E staining of pancreatic sections from indicated mice. **d**, **e** Immunostaining for tdT, zsGreen and CK19 (**d**) or Amylase (**e**) on sections collected from the tail region of PDL pancreas. Yellow arrows, tdT^+^ duct cells. Yellow arrowheads, tdT^+^ acinar cells. **f**, **g** Immunostaining for tdT, zsGreen and CK19 (**f**) or Amylase (**g**) on sections collected from the head region of PDL pancreas. White arrowheads, zsGreen^+^ duct cells. Yellow arrowheads, tdT^+^ acinar cells. **h** Quantification of the percentage of duct cells expressing zsGreen or tdT and acinar cells expressing zsGreen or tdT in the tail and head regions of pancreas from *Tnni3-Dre;CK19-CreER;IR1* mice after PDL-induced injury. Data are means ± SEM. *n* = 5. **i** Diagram illustrating that a subset of new duct cells in the injury region is derived from tdT^+^ acinar cells after PDL. Scale bars, yellow, 1 mm; white and black,100 μm. Each image is representative of 5 individual samples.

PDL triggers a strong inflammatory response and widespread pancreatic acinar cell death in the ligated tail of the pancreas^13,19^. A previous study showed that *CAII*-expressing pancreatic duct cells act as pancreatic progenitors in response to PDL-induced injury^11^. We next performed PDL in *Tnni3-Dre;CK19-CreER;IR1* mice after tamoxifen induction and washout period, and collected pancreatic tissues for analysis 2 weeks after PDL (Fig. 5a). By whole-mount fluorescent imaging, we found a loss of tdT signal in the ligated tail compared with the head of the pancreas (Fig. 5b). By hematoxylin and eosin (H&E) staining of pancreatic sections, we observed severe inflammation, dramatic acinar cell regression and an increase of ductal cells in the exocrine tissues of the tail region of the pancreas compared to the head region (Fig. 5c). Contrary to a previous study^11^, immunostaining for tdT, zsGreen and CK19 or Amylase on tissue sections revealed that all surviving acinar cells were tdT^+^ZsGreen^–^ after PDL injury (Fig. 5d, e), indicating that the pancreas does not exhibit ductal-to-acinar cell transdifferentiation after PDL. Notably, we found that a subset of tdT^+^ cells expressed CK19 and no longer expressed the acinar marker Amylase (Fig. 5d, e). And we did not detect any tdT^+^ duct-like cells and zsGreen^+^ acinar cells in the head region of the pancreas, which served as a control (Fig. 5f–h). Taken together, these results indicate that after PDL-induced injury, a small population of tdT-labeled acinar cells survive and integrate into the tubular complex, initiating acinar-to-ductal transdifferentiation (Fig. 5i).

**Fig. 6:**
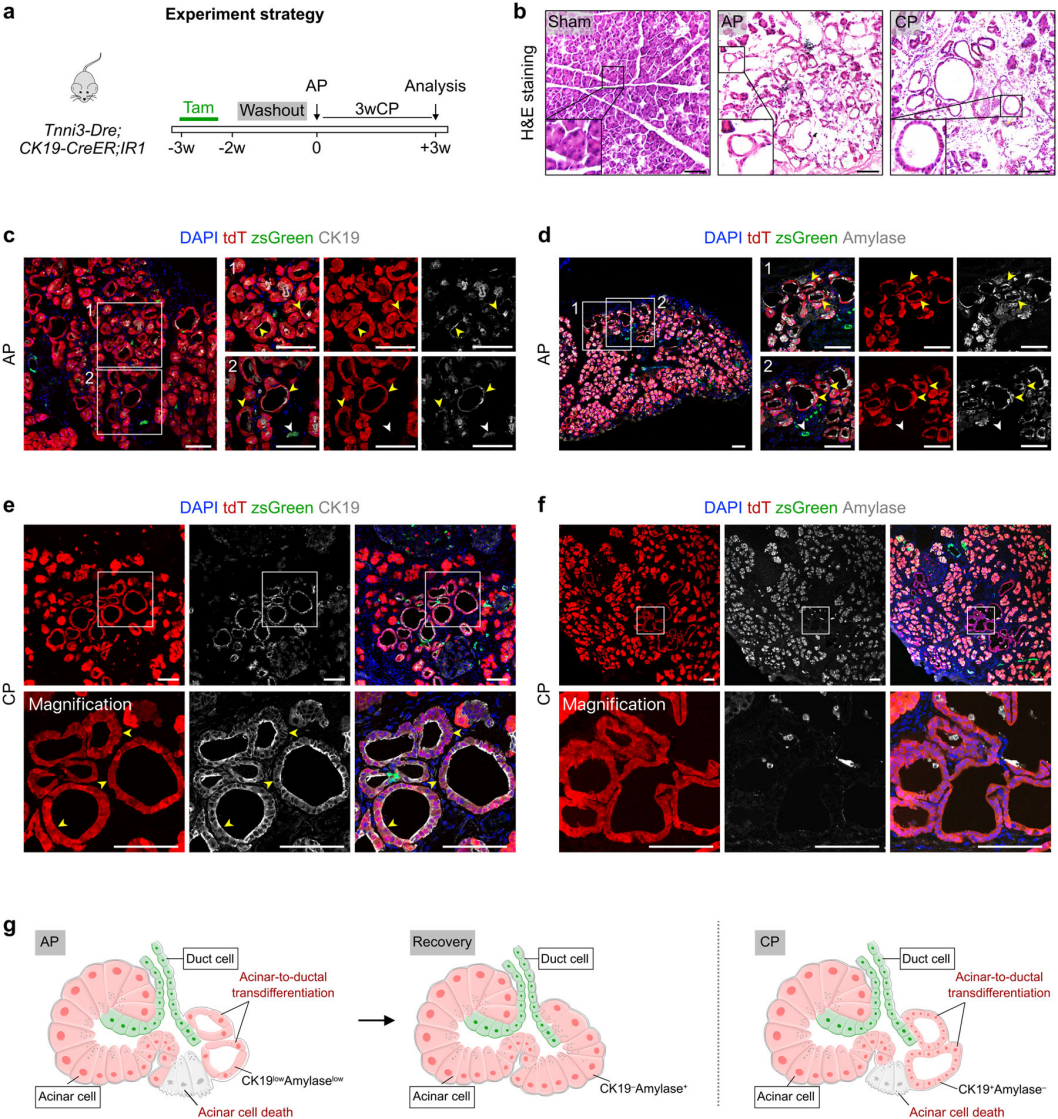
Acinar-to-ductal transdifferentiation occurs during caerulein-induced pancreatitis. **a** Schematic diagram illustrating the experimental strategy for Tam treatment, caerulein-induced AP and CP. **b** H&E staining of pancreatic sections from the indicated mice. **c**–**f** Immunostaining of tdT, zsGreen and CK19 (**c**, **e**) or Amylase (**d**, **f**) on pancreatic sections collected from the indicated mice. White arrowheads, zsGreen^+^ ductal cells. Yellow arrowheads, tdT^+^ ductal cells. **g** Diagram illustrating that a subset of ductal cells is derived from tdT^+^ acinar cells during the caerulein-induced pancreatitis. Scale bars, 100 μm. Each image is representative of 5 individual samples.

Pancreatitis is a common, painful inflammatory disease, and most often arises from inflammation of the exocrine pancreas^2^. To further assess whether acinar cells contribute to ductal cells during pancreatitis, we injected caerulein intraperitoneally to *Tnni3-Dre;CK19-CreER;IR1* mice to induce acute pancreatitis (AP), and collected pancreatic tissues 48 h (the acute pancreatitis phase) after injury for analysis (Fig. 6a). As an analogue of the hormone cholecystokinin, caerulein triggers rapid pancreatitis in mammals and a full rapid spontaneous recovery^2,14^. By H&E staining of pancreatic sections, we found a marked increase of tubular cells in the AP mice (Fig. 6b). Immunostaining for tdT, zsGreen, CK19 or Amylase showed that a subpopulation of tdT^+^ cells lost their normal architecture and gained a duct-like cell phenotype, characterized by degranulation and morphological change (Fig. 6c, d). However, these tdT^+^ cells expressed very weak CK19 and Amylase, indicating these cells were not fully functional ductal cells (Fig. 6c, d), which is consistent with a previous study^15^. We also performed single-cell RNA sequencing on pancreas after caerulein-induced acute pancreatitis. Compared with the adult normal pancreas in previous study^32^, the expression levels of *Krt19* and *Sox9* were upregulated in the injured pancreatic acinar cells (Supplementary Fig. S9a–d). Comparisons of gene expression between *Krt19*^high^ acinar cells and *Krt19*^low^ acinar cells in mice with pancreatitis highlighted enriched pathways for cytoplasmic translation, chemical carcinogenesis, proteasome degradation, tight junction, actin cytoskeleton organization, positive regulation of organelle organization and negative regulation of apoptotic signaling pathway, suggesting that caerulein treatment may endow these *Krt19*^high^ acinar cells with particular properties, e.g., loss of acinar cell functions, enhanced cell–cell junction organization and inflammatory response (Supplementary Fig. S9e).

After pancreatitis subsides, pancreatic acinar cells regenerate spontaneously and robustly, and we observed a striking loss of tdT^+^ duct-like cells and the full recovery of pancreatic acinar cells (Supplementary Fig. S9f–i). We believe that these new acinar cells may originate from pre-existing acinar cells or by “re-differentiation” from these duct-like acinar cells. We also performed long-term caerulein treatment for induction of CP (Fig. 6a). We found some tdT^+^ tubular complexes lined by a monolayer of duct-like cells. However, unlike acute pancreatitis mice, these tubular complexes in CP were lined by many small cells with tight-junctions (Fig. 6b). Meanwhile, these acinar cell-derived tdT^+^ cells were highly expressing CK19 but did not maintain expression of the acinar cell marker Amylase anymore (Fig. 6e, f). These data provide evidence that acinar-to-ductal cell transdifferentiation transiently occurs during the injury phase of caerulein-induced pancreatitis but not during the recovery phase (Fig. 6g).

### Genetic ablation of acinar cells induces duct-to-acinar transdifferentiation

Under physiological conditions and in non-genetically manipulated injury models for inducing acinar cell regeneration, we did not detect zsGreen^+^ acinar cells derived from duct cells in the pancreas. To explore the transdifferentiation potential of pancreatic ductal cells, we generated a knock-in mouse line, *Cela1-DTR*, in which a diphtheria toxin receptor (DTR) is regulated by the acinar cell-specific Elastase promoter (Supplementary Fig. S10a). By whole-mount fluorescent imaging we found a normal morphology of the pancreas in adult *Cela1-DTR* mice (Supplementary Fig. S10b). Immunostaining for Amylase and DTR on pancreatic sections of *Cela1-DTR* mice showed co-expression of DTR and Amylase in pancreatic acinar cells (Supplementary Fig. S10c, d). DT treatment led to a significant reduction of E-cad^+^ and Amylase^+^ acinar cells, compared with the control group (Supplementary Fig. S10e–i).

**Fig. 7:**
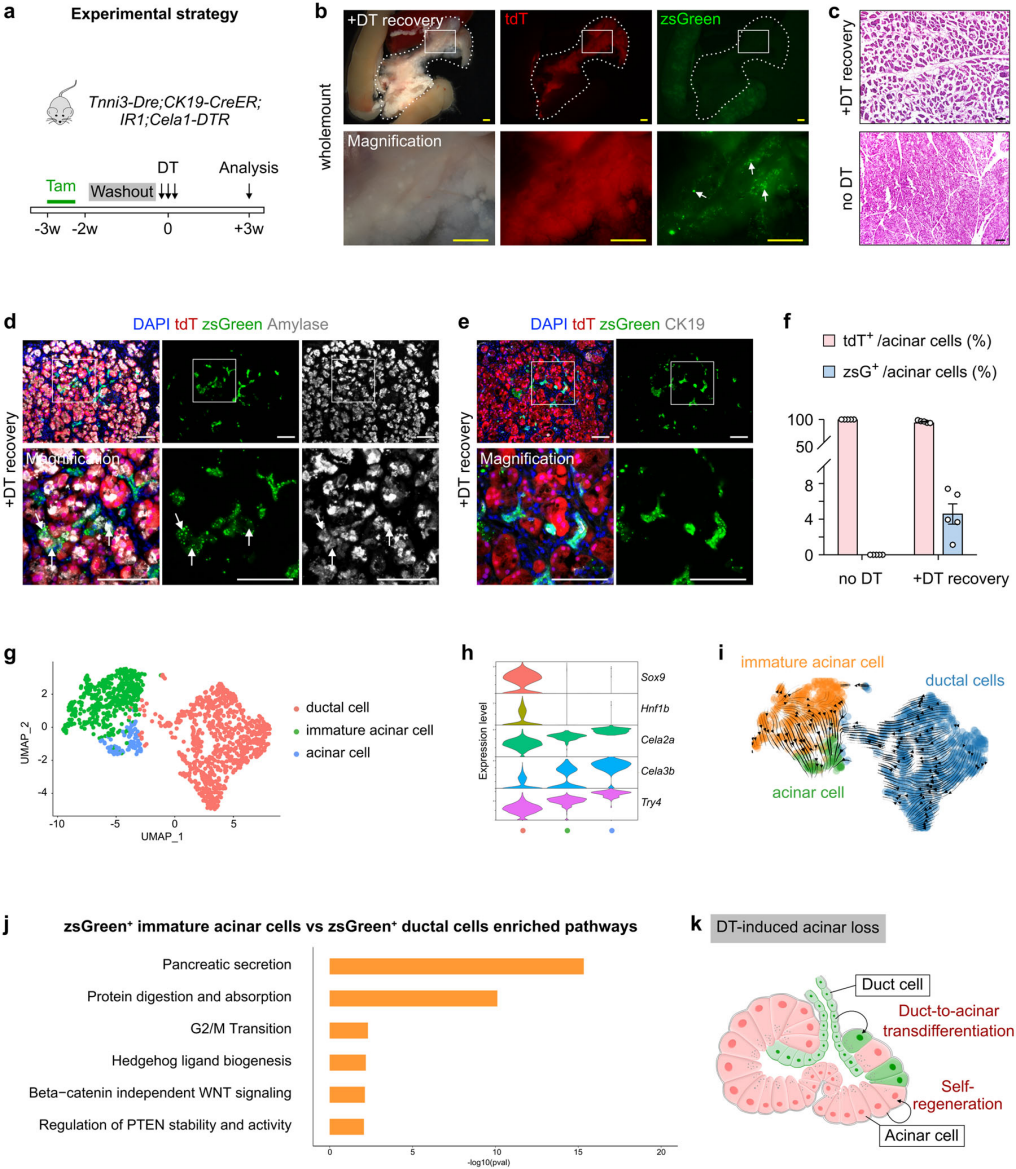
Ductal-to-acinar transdifferentiation occurs after DT-induced acinar cell loss. **a** Schematic diagram illustrating the experimental timeline for cell labeling with Tam, washout, injury by DT treatment and analysis. **b** Whole-mount bright-field and fluorescent images showing zsGreen and tdT expression in the pancreas from *Tnni3-Dre;CK19-CreER;IR1;Cela1-DTR* mice 3 weeks after DT treatment. **c** H&E staining on pancreatic sections from the indicated mice. **d**, **e** Immunostaining for tdT, zsGreen and Amylase (**d**), or CK19 (**e**) on pancreatic sections collected from the indicated mice. White arrows, zsGreen^+^ acinar cells. **f** Quantification of the percentage of acinar cells expressing zsGreen or tdT from the indicated mice. Data are means ± SEM; no DT group, *n* = 5; +DT recovery group, *n* = 5. **g** UMAP visualization of the zsGreen^+^ epithelial cell clusters. **h** UMAP plots showing expression of indicated genes. **i** UMAP embedding of RNA velocity of zsGreen^+^ pancreatic epithelial cells from *Tnni3-Dre;CK19-CreER;IR1;Cela1-DTR* mice after recovery. **j** Selected GO terms enriched in zsGreen^+^ immature acinar cells compared with zsGreen^+^ ductal cells. **k** Diagram illustrating that a subset of new acinar cells is derived from zsGreen^+^ ductal cells after DT-induced acinar cell loss. Scale bars, yellow, 1 mm; white and black,100 μm. Each image is representative of 5 individual samples.

Having successfully generated the *Cela1-DTR* line, we crossed it with *Tnni3-Dre;CK19-CreER;IR1* mice to trace pancreatic acinar and ductal cells after genetic ablation of acinar cells by DT (Fig. 7a). After 3 weeks of recovery, the DT-treated group showed a significant recovery of pancreatic exocrine tissues (Fig. 7b). By H&E staining, we found that acinar cells reappeared after the recovery period (Fig. 7c). A study using a similar strategy previously showed that the ductal cell is the major source for endocrine and acinar cell after DT-induced pancreatic damage^33^. However, our observation that ~95% of regenerated acinar cells originated from the pre-existing tdT^+^ acinar cells that may have escaped from DT treatment and survived is different from the earlier report. This difference may be due to the different genetic mouse models used. Indeed, we found that a subset of zsGreen^+^ cells expressed the acinar cell marker Amylase (Fig. 7d–f), indicating that the pancreatic ductal cells can transdifferentiate to acinar cells after genetic ablation of acinar cells. We did not find the tdT^+^ ductal or duct-like cells in this model (Fig. 7e).

To further explore the transdifferentiation potential of pancreatic ductal cells, we performed single-cell RNA sequencing of zsGreen^+^ pancreatic epithelial cells from *Tnni3-Dre;CK19-CreER;IR1;Cela1-DTR* mice. The expression levels of several acinar markers including *Cela2a*, *Cela3b*, and *Try4* in a subpopulation of acinar cells were lower than those in mature acinar cells, and we defined this subpopulation as immature acinar cells (Fig. 7g, h). We used UMAP to embed RNA velocity of zsGreen^+^ pancreatic epithelial cells from *Tnni3-Dre;CK19-CreER;IR1;Cela1-DTR* mice. The dynamic trajectories indicated a transition progression from ductal cells to immature acinar cells and then to mature acinar cells (Fig. 7i). Gene Ontology (GO) analysis revealed that the upregulated genes in immature acinar cells compared with ductal cells were associated with pancreatic secretion, protein digestion and absorption, G2/M transition, Hedgehog ligand biogenesis, beta-catenin independent WNT signaling and regulation of PTEN stability and activity. Above data indicated that ductal-to-acinar transdifferentiation might be associated with Hedgehog, WNT, and PTEN-related signaling pathways (Fig. 7j). In summary, pancreatic ductal epithelial cells could contribute to the regeneration of acinar cells after DTR-mediated pancreatic injury in adult mice (Fig. 7k).

## Discussion

**Fig. 8:**
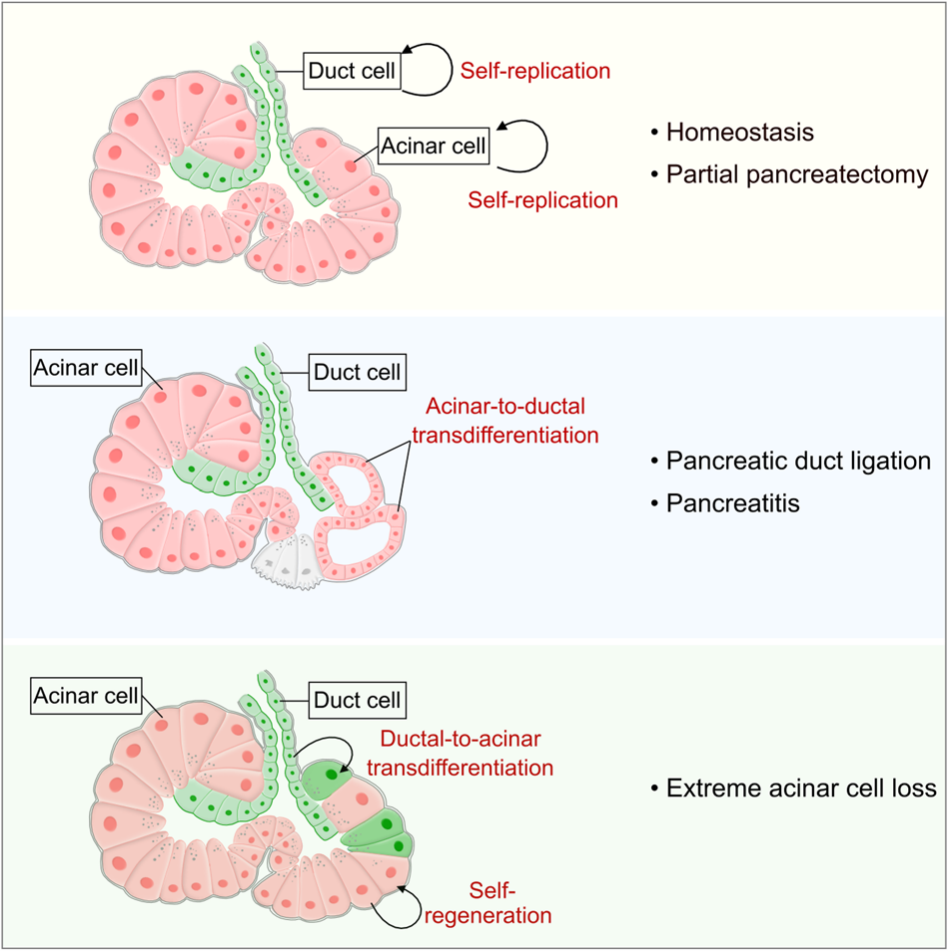
The cell fate transdifferentiation between pancreatic acinar cells and duct cells in adults. Cartoon showing a dual recombinase-mediated genetic lineage tracing of pancreatic acinar cells and duct cells simultaneously.

Taken together, this work illustrates a dual recombinase-mediated genetic approach for simultaneously tracing pancreatic acinar and ductal cells with two distinct indelible genetic markers in the adult pancreas. During homeostasis and PPX-induced pancreatic regeneration, acinar cells and ductal cells maintain their own cell fate through self-proliferation without any noticeable lineage transdifferentiation between the two cell types. Additionally, we designed our study to calculate the turnover rates of pancreatic exocrine lineages during physiology by using the ProTracer system. After PDL- and caerulein-induced injuries, we found that acinar-to-ductal transdifferentiation occurs in the inflammatory environment. On the other hand, when acinar cells were genetically ablated, pancreatic ductal cells could transdifferentiate into new acinar cells. These results demonstrate that acinar cells and ductal cells in the pancreatic exocrine gland undergo lineage conversions under certain pathological conditions (Fig. 8).

Both pancreatic acinar cells and ductal cells originate from the common multipotent pancreatic progenitor cells during embryonic development^1,8–10^. The controversies regarding the lineage conversions between acinar cells and ductal cells in the adult pancreas in previous studies could be partly due to the nonspecific labeling by the proposed cell markers. It is worth noting that the weak expression of ductal cell marker genes driving Cre below the antibody detection sensitivity may unexpectedly induce homologous recombination in some acinar cells, thus resulting in false-positive results. The advantage of our approach here is specific labeling of these two exocrine lineages. As *CK19-CreER* could label a subset of acinar cells in addition to ductal cells, combination of *Tnni3-Dre* eliminates the unwanted acinar cell labeling by *CK19-CreER* through interleaved reporter strategy.

In addition, the studies arguing against the contribution of pancreatic ductal cells or stem cells to acinar cells mainly employed a pulse-chase strategy for studying acinar cells and reported that the labeling percentage of acinar cells was not diluted significantly^13,14^. However, such dilution quantification based on a pulse-chase strategy is not sufficiently sensitive to detect a very small contribution of acinar cells from ductal cells. Due to these technical limitations, the potential of cell lineage conversion between pancreatic acinar cells and ductal cell requires further investigation. Our approach differs from the pulse-chase strategy, as the dual-tracing approach enables detection of very small number of cell lineage conversions based on positive fluorescent reporter detection rather than on dilution.

It has been reported that *Bmi1*^*+*^ acinar cells are a subpopulation capable of self-renewal and proliferation during tissue homeostasis and after injury^16^. Wollny et al. demonstrated that adult pancreatic acinar cells are heterogeneous using single-cell RNA sequencing, and they found a progenitor-like acinar cell subpopulation that could be the source of new acinar cells^34^. Furthermore, Westphalen et al. found that pancreatic *Dclk1*^*+*^ cells are the quiescent progenitor cells involved in tissue regeneration and tumorigenesis^35^. Conversely, Lodestijn et al. used an unbiased continuous clonal labeling strategy recently and found that all acinar cells have an equal probability to contribute to acinar cell renewal during homeostasis^36^. Thus, at this point, the question whether a subpopulation of pancreatic acinar cells is able to contribute to new acinar cells in the adult pancreas remains unclear and needs further investigation.

Together, the system and information provided in this study could potentially provide clinically relevant information leading to the design of new therapeutic options to treat pancreatic-related pathologies, while also allowing for the further investigation of the cellular and molecular mechanisms of pancreatic exocrine cell plasticity in different pathological conditions, including cancers.

## Materials and methods

### Mice

All mouse experiments were carried out in accordance with the guidelines of the Institutional Animal Care and Use Committee (IACUC) at the Center for Excellence in Molecular Cell Science, Shanghai Institute of Biochemistry and Cell Biology, Chinese Academy of Science. All mice were maintained on a C57BL6/ICR mixed background, housed in standard cages and maintained on a 12 h light/dark cycle and fed with normal diet. *CK19-CreER*, *Tnni3-Dre*, *R26-iCre*, *IR1, R26-DreER, Ki67-CrexER* and *R26-RSR-LSL-tdT* mouse lines were described previously^24,25,28,30,37^. *Cela1-DTR* was generated using CRISPR/Cas9 by Shanghai Model Organism Center, Inc. (SMOC). Briefly, a cDNA encoding DTR was targeted into the translational stop codon of the *Cela1* gene, following endogenous *Cela* by the self-cleaving peptide 2 A sequence. Genomic DNA was extracted from the mouse tail snip. Tissues were lysed using lysis buffer (100 mM Tris HCl, pH 7.8, 0.2% SDS, 200 mM NaCl, 5 mM EDTA and 100 μg/mL proteinase K) over 6 h at 55 °C. The mixture was centrifuged at the 13,000× *g* for 8 min to obtain supernatant. Then the DNA was precipitated by isopropanol and washed one time with 70% ethanol and dissolved in ddH_2_O. PCR primers were designed to distinguish the correctly targeted knock-in allele (*Cela1-DTR* 5’mut&wt-F: TGCTTGTCTTGGCAAACACTGATCT, 5’mut-R: CCGGGTTGCTGGTTCCAGCAG, 5’wt-R: GAAAGGAAGAGCCAGGCCATGG). Both male and female mice were randomly allocated to control and experimental groups. Tamoxifen (Sigma, T5648) was dissolved in corn oil (20 mg/mL) and administered by gavage (0.2 mg/g) at the adult stage. For *CK19-CreER;R26-tdT*, *Tnni3-Dre;CK19-CreER;IR1*, and *R26-DreER;Ki67-CrexER;R26-RSR-LSL-tdT* mice, tamoxifen treatment was performed for three times in 5 days. Doxycycline (Sigma, D9891) was dissolved in sterile H_2_O (2 mg/mL) to feed for 1 week and also in sterile H_2_O (20 mg/mL) to administrate by gavage (0.2 mg/g) for five times. DT was dissolved in PBS (1 ng/μL) and introduced by intraperitoneal injection (10 ng/g/day) per mouse, three times each, to induce pancreatic acinar cell loss.

### PPX

PPX was carried out as previously described^29^. Adult mice were anesthetized with 2% isoflurane gas in the sealed chamber and transferred onto a heat pad. Then anesthesia was maintained by the inhalation of isoflurane. After removal of the abdominal fur, a midline abdominal incision was made. The mesenteric connections to the stomach, small intestine, and retroperitoneum were partially removed. The splenic lobe of the pancreas was moved and then ~50% pancreatic tissue, bordered by the spleen and stomach, but not including the small flap of pancreas, was removed. The mouse from sham group underwent laparotomy. Then the mouse was placed in individual cage and received a standard laboratory chow diet and tap water postoperatively.

### PDL

PDL was carried out as previously described^29^. Briefly, adult mice were anesthetized with 2% isoflurane gas in the sealed chamber and transferred onto a heat pad to maintain body warm. Then anesthesia was maintained by the inhalation of isoflurane. After removal of the abdominal fur, a midline abdominal skin and muscle incision was made. The head of the pancreas and the duodenum were lifted off the retroperitoneum. The pancreatic duct at the left side of the portal vein, which separates the splenic and gastro-duodenal lobes was carefully ligated. The mouse from sham group underwent laparotomy. Then the mouse was placed in individual cage and received a standard laboratory chow diet and tap water postoperatively.

### Pancreatitis

Caerulein**-**induced AP and CP were carried out as previously described^15^. Briefly, adult mice were received with 6 hourly intraperitoneal injections of caerulein (50 μg/kg) (MCE, HY-A0190) every day for 2 days after a fasting period of 12 h to induce AP. The pancreas was harvested 48 h and 2 weeks (for recovery) after the last injection. CP was induced by three series of injection every week for 3 weeks. The pancreas was harvested 72 h after the last caerulein injection.

### Whole-mount fluorescence microscopy

The collected pancreatic tissues at the indicated time points were washed three times (for 10–15 min each time) with phosphate-buffered saline (PBS, pH 7.4) and placed on an agar gel in a petri dish. The whole-mount bright-field and fluorescent images were taken using the Zeiss stereo microscope (AxioZoom V16).

### Immunofluorescent staining

Immunofluorescent staining was performed as previously described^29^. Briefly, pancreatic tissues were collected in cold PBS and fixed in 4% paraformaldehyde (PFA) for 1 h at 4 °C. Then the tissues were washed three times (for 10–15 min each time) with PBS at room temperature and dehydrated in 30% sucrose overnight at 4 °C. The tissues were then embedded in optimum cutting tissue (OCT, Sakura) for 1 h at 4 °C, and frozen in block with OCT and sectioned using cryosection machine (Thermo Fisher, HM525). Ten micrometer thick cryosections were collected on slides, washed 15 min with PBS, incubated in 5% normal donkey serum (NDS) in PBST (PBS with 0.1% Triton X-100) for 30 min at room temperature and then incubated with primary antibodies (diluted in 2.5% NDS diluted in PBST) overnight at 4 °C. All primary antibodies were commercially available reagents: tdT (Rockland, 600-401-379, 1:1000), tdT (Rockland, 200-101-379, 1:1000), zsGreen (Clontech, 632474, 1:1000), E-cad (Cell Signaling, 3195, 1:500), Amylase (Sigma, A8273, 1:500), Cytokeratin 19 (Developmental Studies Hybridoma Bank, TROMA-III, 1:200), Cytokeratin 19 (Abbomax, 602-670, 1:500), PDGFRa (R&D, AF1062, 1:500), VE-cadherin (R&D, AF1002, 1:100), Lymphatic vessel endothelial hyaluronan receptor 1 (Abcam, ab14917, 1:500), human HB-EGF (DTR, R&D, AF-259-NA, 1:200). After washing three times with PBS, signals were developed with the secondary Alexa fluorescent conjugated antibodies (Invitrogen, 1:1000) for 0.5 h at room temperature and counterstained with DAPI (1:1000). After this, slides were washed three times with PBS and mounted with fluorescence-protecting mounting medium (Vector Lab). Images were obtained using Nikon confocal microscope (Nikon A1), Olympus confocal microscope (Olympus FV1200) or Zeiss confocal microscope (Zeiss LSM880) and image data were analyzed by the ImageJ (NIH) software.

### H&E staining

H&E staining was performed as previously described^29^. Briefly, 10 μm-thick cryosections were washed for 15 min with PBS and incubated in Hematoxylin A solution for 3 min, washed three times with running tap water, rinsed in 1% concentrated hydrochloric acid diluted in 70% ethanol for 1 min and washed three times with water. The sections were then incubated in 1% ammonia water for 1 min, washed three times with water, stained with Eosin-Y solution for 8–10 sections, dehydrated in a series of ethanol and xylene, and lastly, mounted with resinous medium. All images were acquired using an Olympus microscope (Olympus, DP72).

### Southern blot

Southern blot analysis was performed as previously described^28^. Briefly, genetic DNA was isolated from mouse pancreatic tissues of indicated mice. The Cre-specific oligonucleotide probes were PCR-amplified from Ki67-CrexER vector and labeled with digoxigenin-dUTP by a PCR DIG probe synthesis Kit (Roche diagnostics, GmbH, Germany). The primer pairs for DNA probes amplification were Probe-F: ACGTATAGCCGAAATTGCCAGGA and Probe-R: CAGAGTCATCCTTAGCGCCGTAA. 30 μg DNA was digested with *Apa*LI restriction enzyme (NEB, Massachusetts, US) overnight at 37 °C, and then separated on the 0.8% agarose gel and blotted with the positively charged nylon membrane (Roche diagnostics, GmbH, Germany). According to the protocol of the DIG easy hybridization Kit (Roche diagnostics, GmbH, Germany), the DNA was exposed to UV, prehybridized and hybridized. Then the probes were washed and blocked. The detection of hybrid products was performed according the CDP-Star (Roche diagnostics, GmbH, Germany) and the membrane was exposed to X-ray at room temperature for 20 min.

### Cell isolation and FACS

Pancreatic tissue isolation was performed as previously^36^ with minor modifications. Briefly, pancreas was minced into small pieces, and transferred to 3 mL 0.5 mg/mL Collagenase P (Roche, 11213873001) dissolved in HBSS together with 5 mM glucose. Then the digestion medium was put into the water bath at 37 °C 5 min for acclimatization and gently shaking another 5 min for digestion. To stop the digestion, 10 mL DMEM containing 1% FBS was added to the medium. Tissues were pipetted up and down for further dissociation and filtered by the 70-μm filter. Then cells were centrifuged for 5 min at 1200 rpm and incubated with 1 mL red blood cell lysis buffer (eBioscience, 00-4333-57) at room temperature for 5 min. 10 mL cold PBS containing 1% FBS was added to stop digestion, followed by centrifugation. 1% Fc in PBS was added for 5 min to block, then the isolated cells were stained with fluorochrome-conjugated antibodies including CD45-APC (eBioscience, 17-0451-82, 1:400), CD31-APC (eBioscience, 17-0311-82, 1:40), CD140a-APC (eBioscience, 17-1401-81, 1:100), CD326-APC (eBioscience, 17-5791-82, 1:200) for 30 min at 4 °C. After washing with cold PBS, cells were resuspended in PBS containing DAPI (1:1000). Then flow cytometry experiments were performed using the Beckman Cytoflex LX or sorted using the Sony MA900 flow cytometer.

### Single-cell RNA sequencing

Single-cell RNA sequencing was performed as previously^27^. The cell suspense was loaded in 10× Chromium controller to generate GEMs, which were further processed into single-cell 3ʹ gene expression libraries using Chromium Single Cell 3ʹ Library Kit (v3.1 Chemistry) or Chromium Single Cell 5ʹ Library Kit. Sequencing was performed on Illumina NovaSeq 6000 PE150 platform. Raw fastq files first were cleaned by Trim Galore with parameter ‘-q 20–phred33–stringency 3–length 20 -e 0.1’. Trimmed fastq files were processed by CellRanger (v6.1.1) pipeline. Further analysis was done by R package Seurat (v4.1.2) with customized parameters. GO enrichment analysis was performed by Metascape using the genes upregulated in *Krt19*^high^ acinar cells versus *Krt19*^low^ acinar cells or *zsGreen*^*+*^ immature acinar cells versus *zsGreen*^*+*^ duct cells. To infer future states of individual cells, we made use of the spliced and unspliced information. We employed scvelo (https://github.com/ theislab/scvelo). The previously normalized and log-transformed data were the starting point to calculate first and second order moments for each cell across its nearest neighbors (scvelo.pp.moments(n_neighbors = 20)). Next, the velocities were estimated and the velocity graph was constructed using the scvelo.tl.velocity() with the mode set to ‘deterministic’ and scvelo.tl.velocity_graph() functions. Velocities were visualized on top of the previously calculated UMAP coordinates with the scvelo.tl.velocity_embedding_stream() function.

### Statistics

All data are presented as means ± SEM. All mice were randomly assigned to experimental or sham groups and all data were obtained from 3–5 individual samples. For all experimental data, calculations were performed using Prism (GraphPad software).

## Supplementary information

Supplementary information

## Data Availability

The SRA project accession numbers of single-cell RNA Sequencing datasets for this project are PRJNA878657 and PRJNA878658. Further information of this study is available from the corresponding author on reasonable request.
