# Supplementary material for: Use of a dual genetic system to decipher exocrine cell fate conversions in the adult pancreas
Source: Cell Discov. 2023 Jan 3;9:1. doi: 10.1038/s41421-022-00485-0 (PMC9810707; doi:10.1038/s41421-022-00485-0)
Supplement: Supplementary file 1 — Supplementary information [file 41421_2022_485_MOESM1_ESM.pdf]

Supplementary Information

# Use of a dual genetic system to decipher exocrine cell fate conversions in the adult pancreas

Huan Zhao, Xiuzhen Huang, Zixin Liu, Liang Lai, Ruilin Sun, Ruling Shen, Yan Li, Lingjuan He, Wenjuan Pu, Zan Lv, Yi Li, Ximeng Han, Xiuxiu Liu, and Bin Zhou

**This PDF file includes**

Supplementary Fig. S1-S10

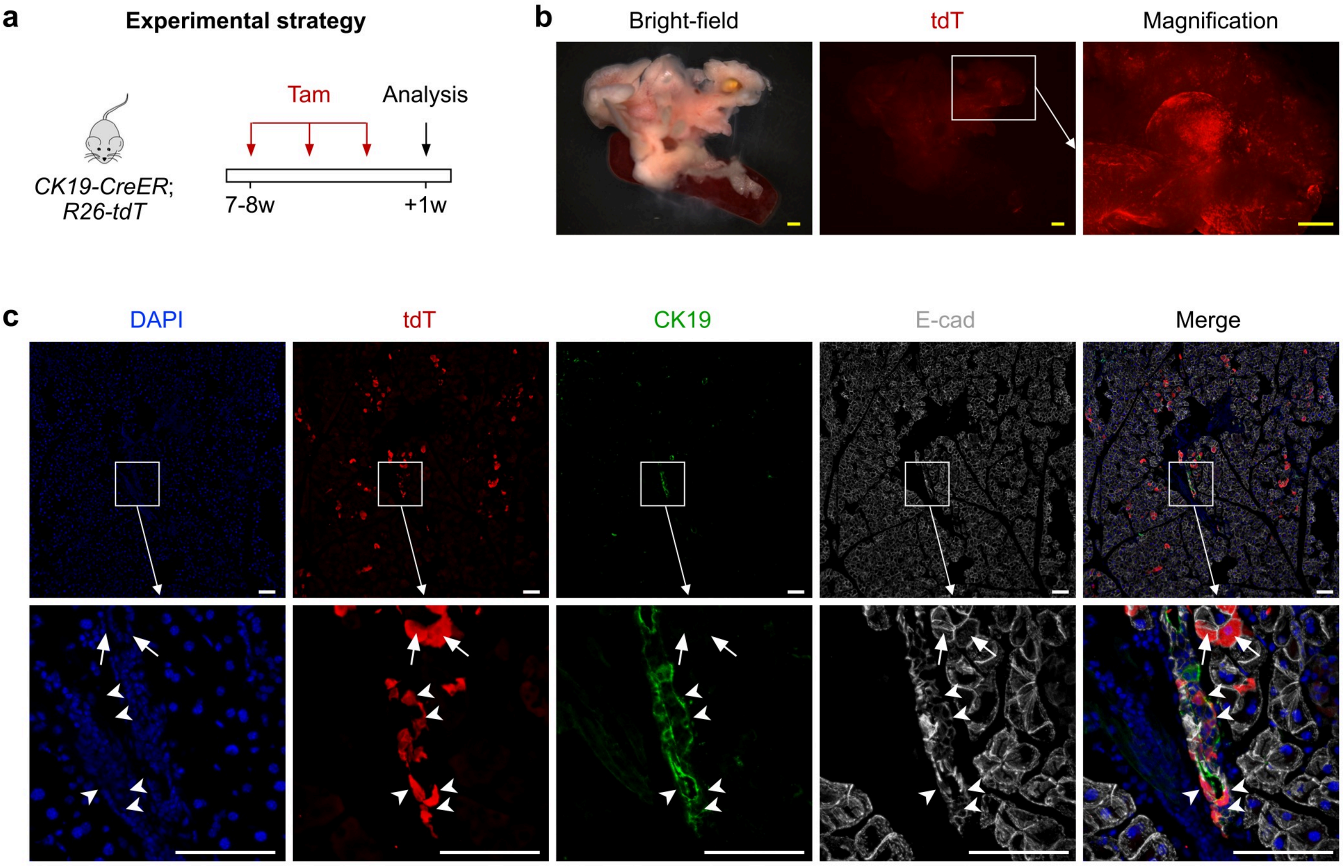


**Supplementary Fig. S1 Characterization of *CK19-CreER* in pancreas. a** Schematic illustrating the experimental strategy for tamoxifen induction and analysis. **b** Whole-mount bright-field and fluorescent images for tdT expression of pancreas. **c** Immunostaining for tdT, CK19 and E-cad on pancreas sections collected from indicated mice. Arrowheads indicating duct cells labeled by *CK19-CreER*. Arrows indicating acinar cells labeled by *CK19-CreER*. Scale bars, yellow, 1 mm; white,100 μm. Each image is representative of 5 individual samples.

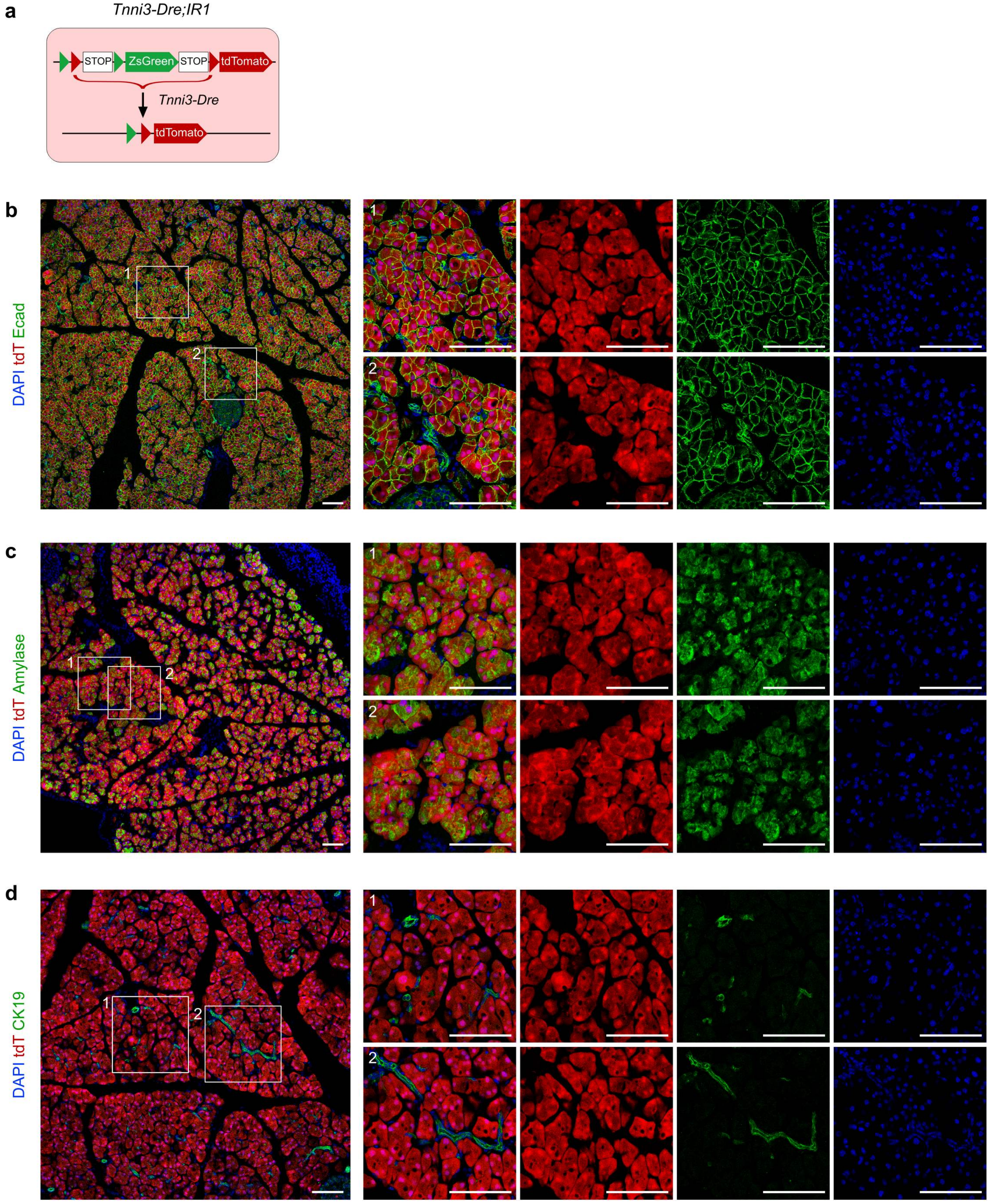


**Supplementary Fig. S2 *Tnni3-Dre;IR1* efficiently labels pancreatic acinar cells but not ductal cells. a** Schematic illustrating the labeling strategy of pancreatic exocrine cells by *Tnni3-Dre;IR1*. **b-d** Immunostaining for tdT and Ecad (**b**) or Amylase (**c**) or CK19 (**d**) on pancreatic sections collected from *Tnni3-Dre;IR1*. Scale bars, 100 μm. Each image is representative of 3 individual samples.

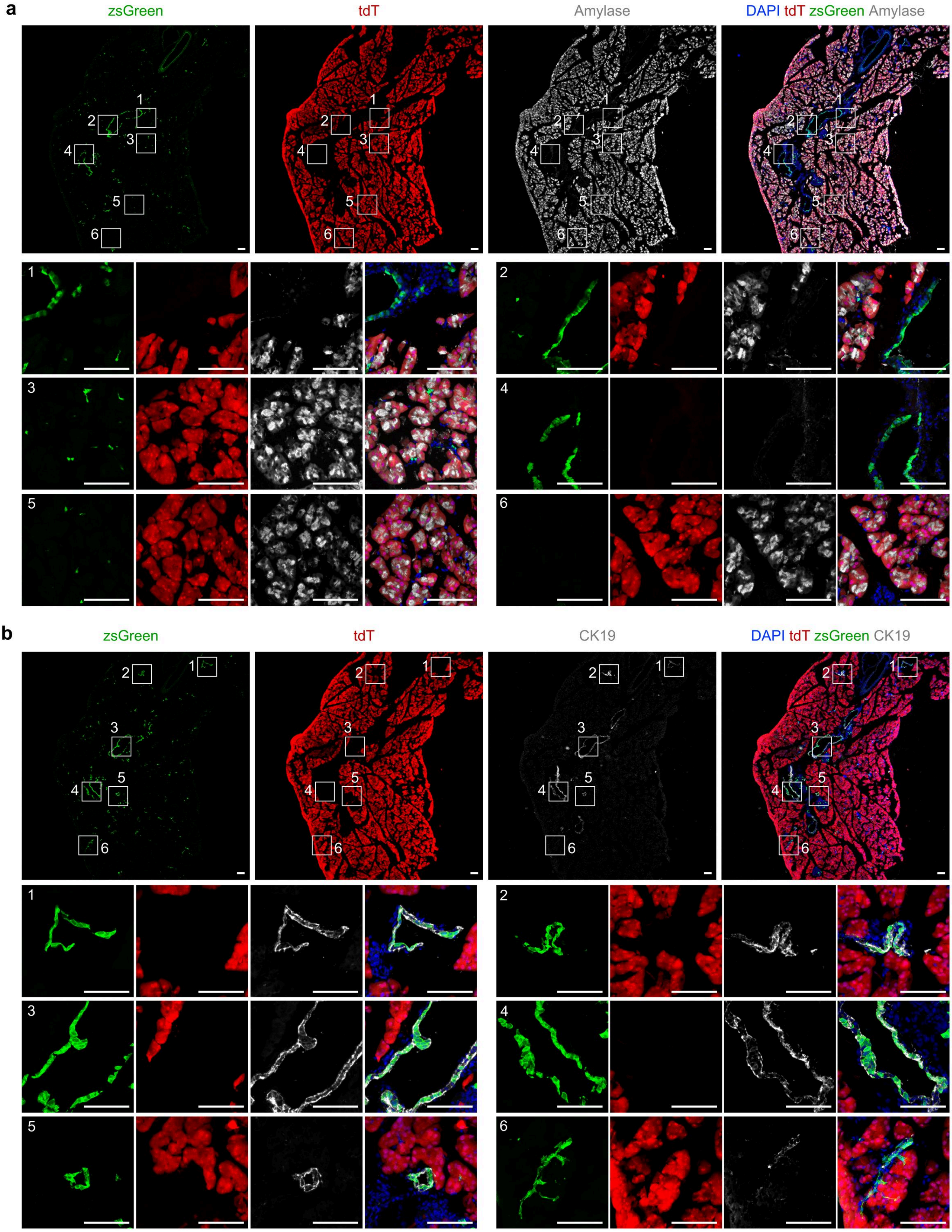


**Supplementary Fig. S3 Almost no zsGreen⁺ acinar and tdT⁺ ductal cells in pancreas of *Tnni3-Dre;CK19-CreER;IR1*. a** Immunostaining for tdT, zsGreen and Amylase on pancreatic sections collected from *Tnni3-Dre;CK19-CreER;IR1* mice after Tam treatment. **b** Immunostaining for tdT, zsGreen and CK19 on pancreatic sections collected from *Tnni3-Dre;CK19-CreER;IR1* mice after Tam treatment. Scale bars, 100 μm. Each image is representative of 3 individual samples.

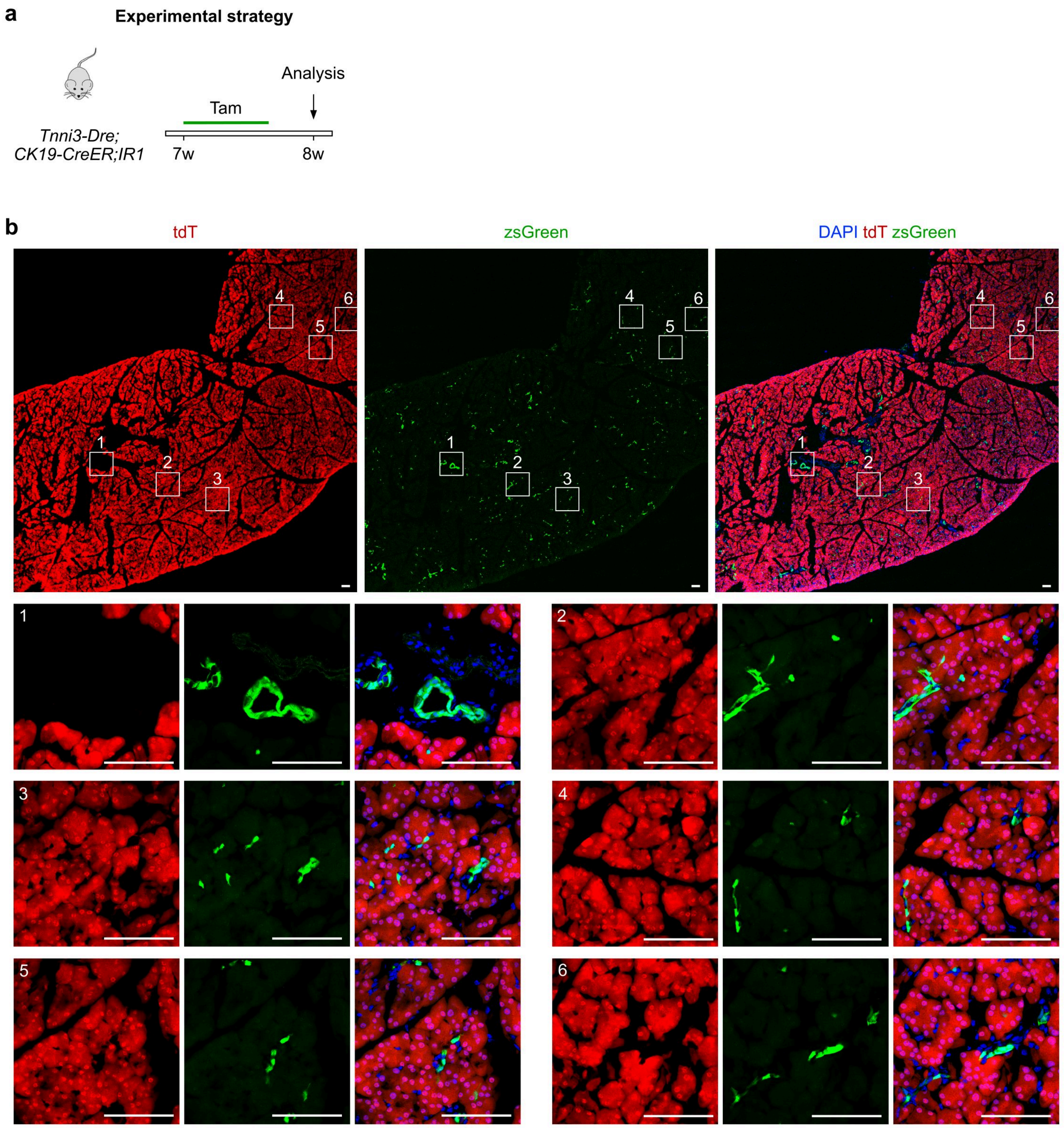


**Supplementary Fig. S4 Almost no tdT⁺zsGreen⁺ cells in pancreas of *Tnni3-Dre;CK19-CreER;IR1*. a** Schematic illustrating the experimental strategy for tamoxifen induction and analysis. **b** Immunostaining for tdT and zsGreen on pancreatic sections collected from indicated mice. Scale bars, 100 μm. Each image is representative of 3 individual samples.

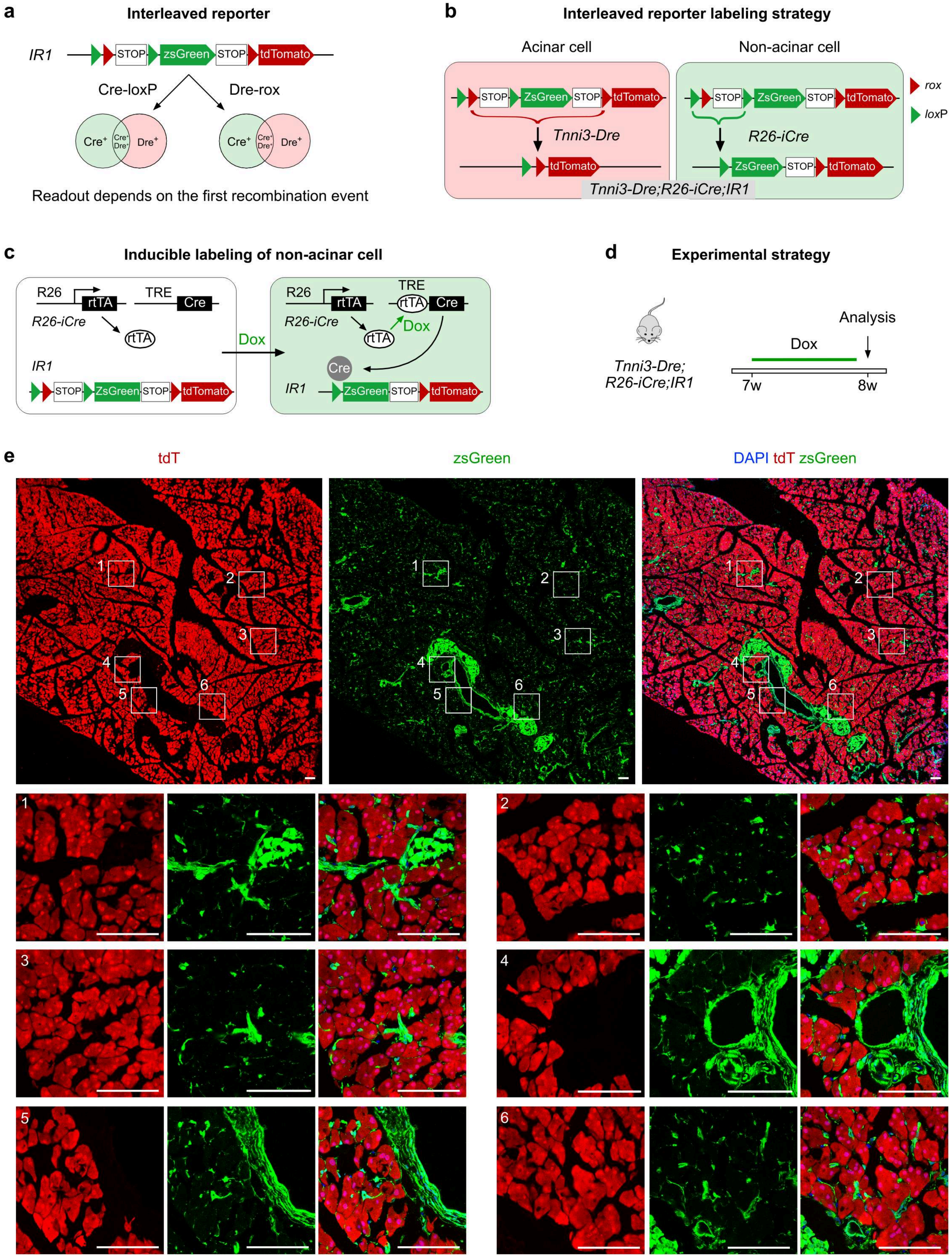


**Supplementary Fig. S5 Almost no tdT⁺zsGreen⁺ cells in pancreas of *Tnni3-Dre;R26-iCre;IR1*. a** Schematic illustrating the labeling strategy of IR1 reporter. The readout of IR1 depends on the first recombination event. **b** Schematic illustrating the labeling strategy of *Tnni3-Dre;R26-iCre;IR1*. **c** Inducible strategy of *R26-iCre*. **d** Experimental strategy for doxycycline (Dox) induction and analysis. **e** Immunostaining for tdT and zsGreen on pancreas sections collected from indicated mice. Scale bars, 100 μm. Each image is representative of 3 individual samples.

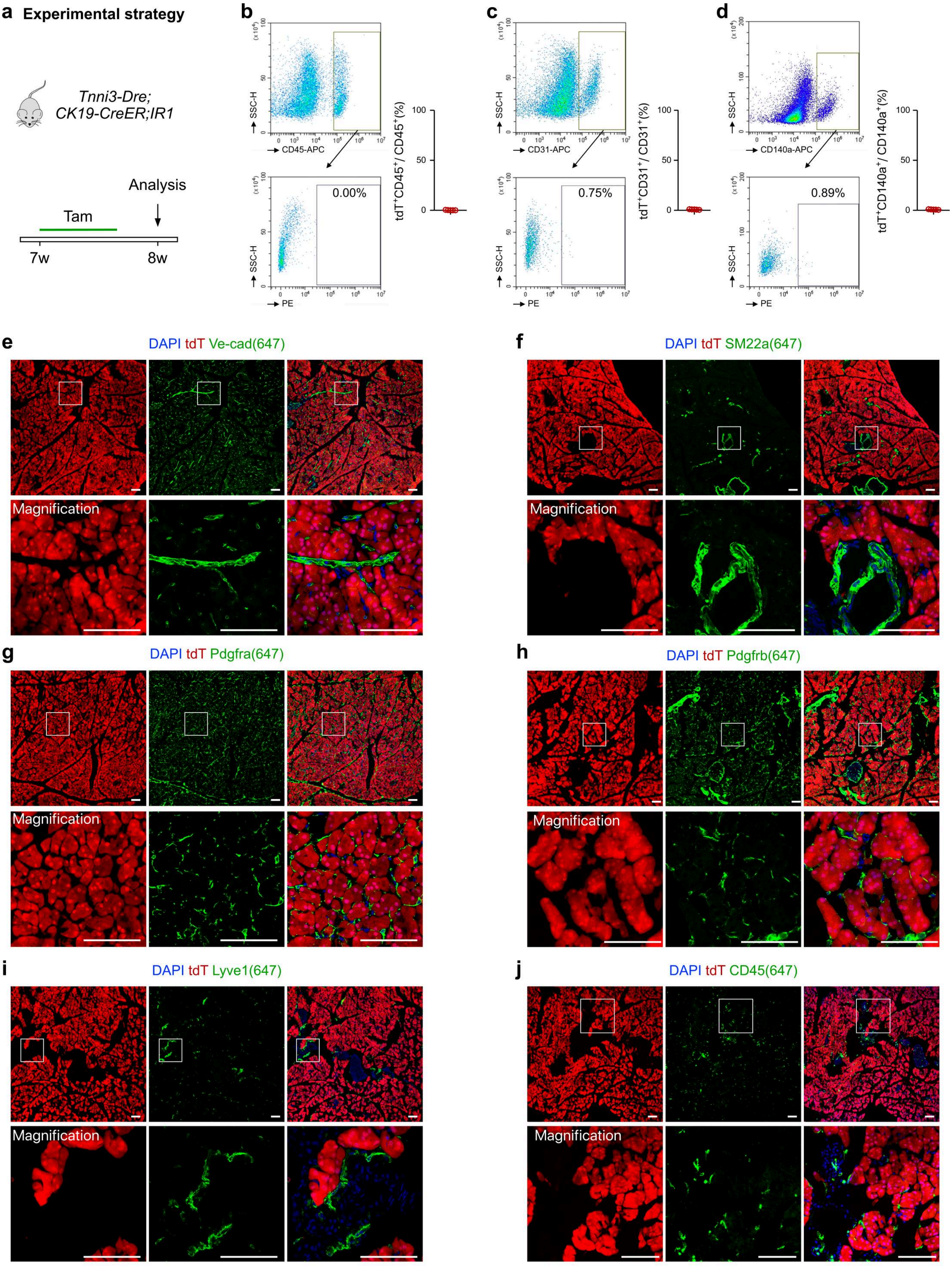


**Supplementary Fig. S6 *Tnni3-Dre* barely labels other cell lineages in pancreas. a** Schematic illustrating the experimental strategy for tamoxifen induction and analysis. **b-d** FACS and quantification of the percentage of $tdT^+$ cells in $CD45^+$ (**b**), $CD31^+$ (**c**), and $CD140a^+$ (**d**) cell lineages. **e-j** Immunostaining for tdT, Ve-cad (**e**), SM22a (**f**), Pdgfra (**g**), Pdgfrb(**h**), Lyve1 (**i**) and CD45 (**j**) on pancreas sections collected from indicated mice. Scale bars, 100 μm. Each image is representative of 5 individual samples.

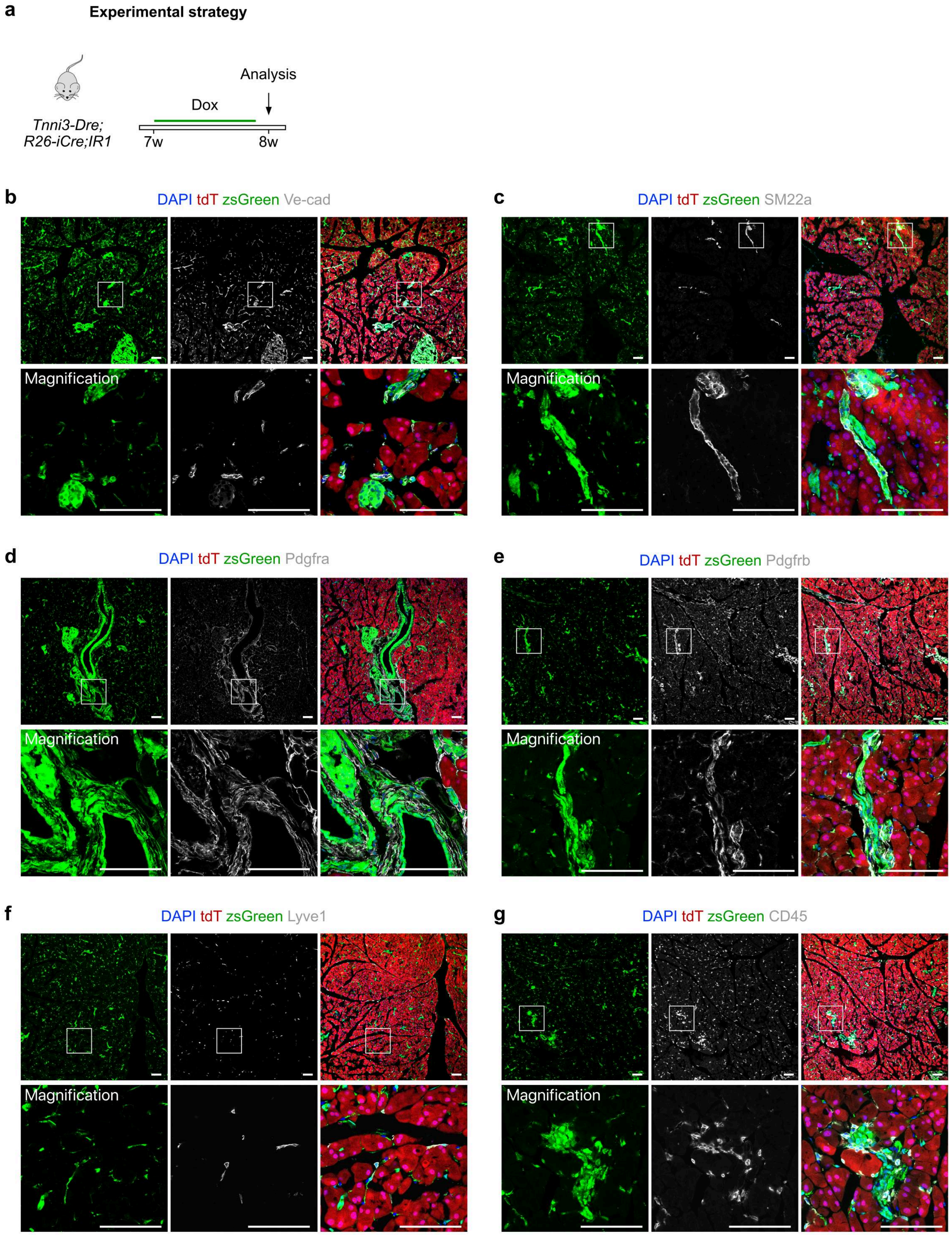


**Supplementary Fig. S7 *R26-iCre* efficiently labels most of cells in pancreas. a** Schematic illustrating the experimental strategy for tDox induction and analysis. **b-g** Immunostaining for tdT, zsGreen Ve-cad (**b**), SM22a (**c**), Pdgfra (**d**), Pdgfrb (**e**), Lyve1 (**f**) and CD45 (**g**) on pancreas sections collected from indicated mice. Scale bars, 100 μm. Each image is representative of 3 individual samples.

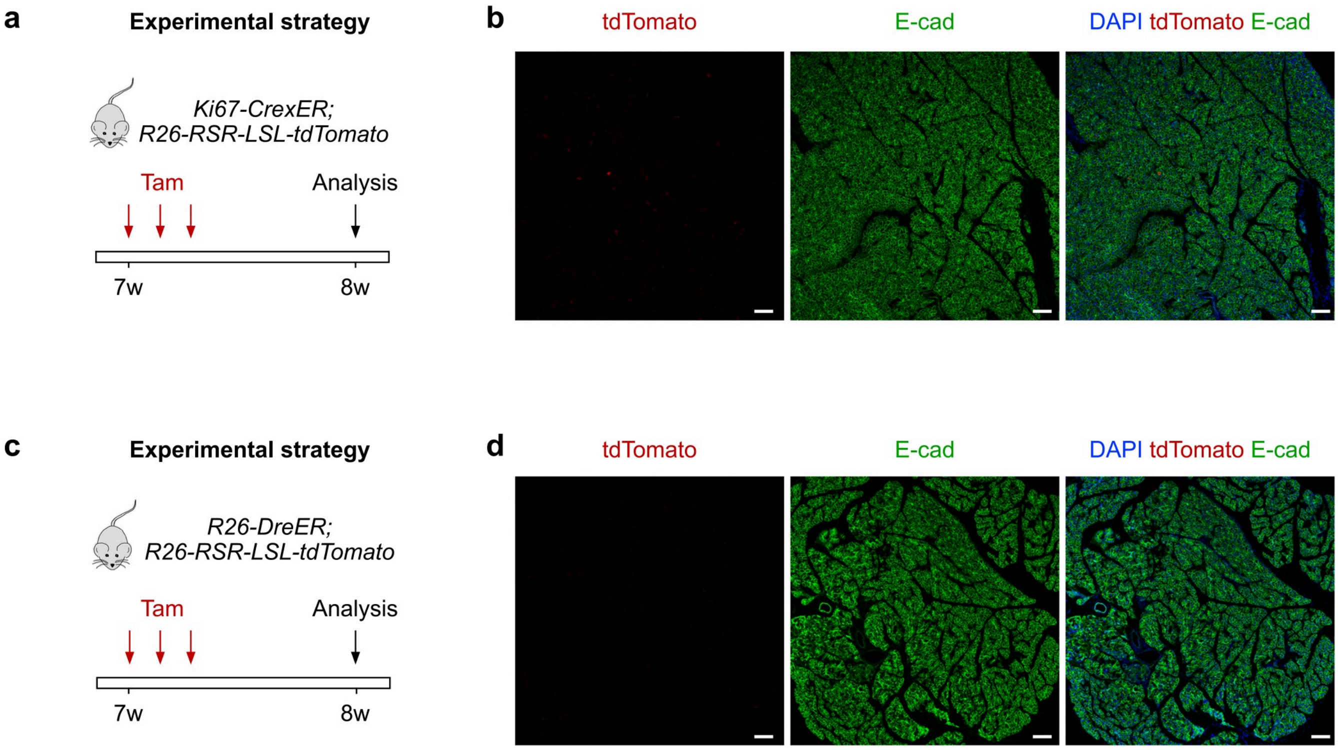


**Supplementary Fig. S8 No detectable Dre-loxP and Cre-rox recombination in pancreas of the Pro-Tracer system. a** Schematic illustrating the experimental strategy for *Ki67-CrexER;R26-RSR-LSL-tdTomato*. **b** Immunostaining for tdT and E-cad on pancreatic sections collected from *Ki67-CrexER;R26-RSR-LSL-tdTomato*. **c** Schematic illustrating the experimental strategy of *R26-DreER;R26-RSR-LSL-tdTomato*. **d** Immunostaining for tdT and E-cad on pancreatic sections collected from *R26-DreER;R26-RSR-LSL-tdTomato*. Scale bars, 100 μm. Each image is representative of 3 individual samples.

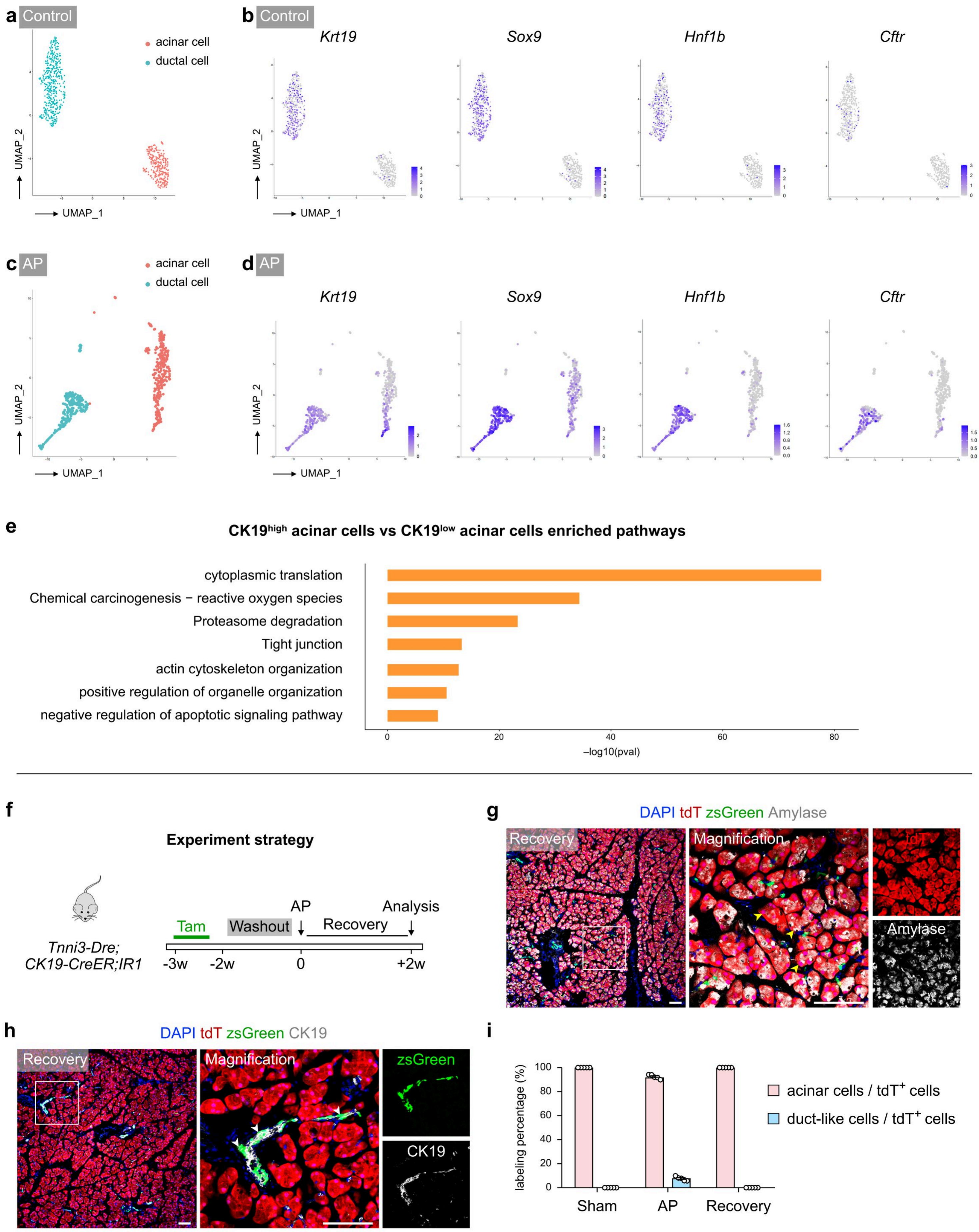


**Supplementary Fig. S9 sc-RNA sequencing analysis of pancreas after caerulein-induced pancreatitis.** **a** UMAP visualization of the epithelial cell clusters in the adult pancreas. Data are retrieved from previous study (Xiaoping Han et al., Cell 2018). **b** UMAP plots showing expression of indicated genes. **c** UMAP visualization of the epithelial cell clusters in the adult pancreas after caerulein-induced pancreatitis. **d** UMAP plots showing expression of indicated genes. **e** Selected GO terms enriched in CK19$^{high}$ acinar cells compared with CK19$^{low}$ acinar cells. **f-g** Immunostaining of tdT, zsGreen and CK19 (**h**) or Amylase (**g**) on pancreatic sections collected from the indicated mice. White arrowheads, zsGreen$^{+}$ duct cells. Yellow arrowheads, tdT$^{+}$ acinar cells. Scale bars, 100 μm. Each image is representative of 5 individual samples.

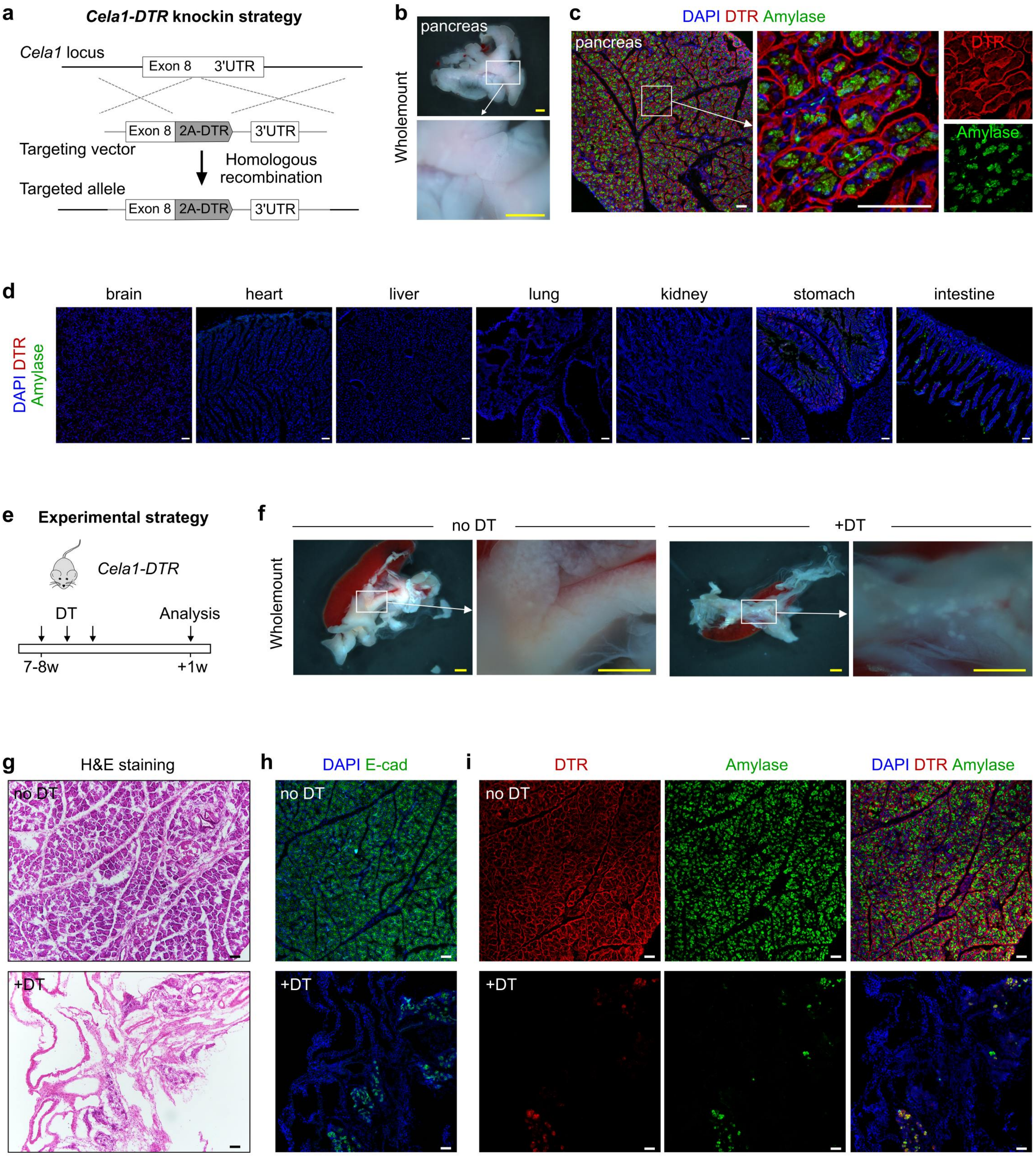


**Supplementary Fig. S10 Generation and characterization of *Cela1-DTR*.** **a** Schematic showing knock-in strategy of *Cela1-DTR.* **b** Wholemount bright-field of pancreas from *Cela1-DTR* mice. **c** Immunostaining for DTR and Amylase on pancreatic sections of *Cela1-DTR* mice. **d** Immunostaining for DTR and Amylase on other organ sections of *Cela1-DTR* mice. **e** Schematic showing experimental strategy of DT injection and analysis. **f** Wholemount bright-field of pancreas from *Cela1-DTR* mice with or without DT treatment. **g** H&E staining on pancreatic sections of *Cela1-DTR* mice with or without DT treatment. **h-i** Immunostaining for E-cad (**h**) or DTR and Amylase (**i**) on pancreatic sections of *Cela1-DTR* mice with or without DT treatment. Scale bars, yellow, 1 mm; white,100 μm. Each image is representative of 5 individual samples.
